# Effect of Telemetric Interventions on Glycated Hemoglobin A1c and Management of Type 2 Diabetes Mellitus: Systematic Meta-Review

**DOI:** 10.2196/23252

**Published:** 2021-02-17

**Authors:** Claudia Eberle, Stefanie Stichling

**Affiliations:** 1 Medicine with Specialization in Internal Medicine and General Medicine Hochschule Fulda–University of Applied Sciences Fulda Germany

**Keywords:** telemedicine, telemetry, diabetes

## Abstract

**Background:**

Diabetes mellitus is a chronic burden, with a prevalence that is increasing worldwide. Telemetric interventions have attracted great interest and may provide effective new therapeutic approaches for improving type 2 diabetes mellitus (T2DM) care.

**Objective:**

The objective of this study was to analyze the clinical effectiveness of telemetric interventions on glycated hemoglobin A_1c_ (HbA_1c_) specifically and T2DM management generally in a systematic meta-review.

**Methods:**

A systematic literature search was performed in PubMed, CINAHL, Cochrane Library, Web of Science Core Collection, and EMBASE databases from January 2008 to April 2020. Studies that addressed HbA_1c_, blood pressure, fasting blood glucose, BMI, diabetes-related and health-related quality of life, cost-effectiveness, time savings, and the clinical effectiveness of telemetric interventions were analyzed. In total, 73 randomized controlled trials (RCTs), 10 systematic reviews/meta-analyses, 9 qualitative studies, 2 cohort studies, 2 nonrandomized controlled studies, 2 observational studies, and 1 noncontrolled intervention study were analyzed.

**Results:**

Overall, 1647 citations were identified. After careful screening, 99 studies (n=15,939 patients; n=82,436 patient cases) were selected by two independent reviewers for inclusion in the review. Telemetric interventions were categorized according to communication channels to health care providers: (1) “real-time video” interventions, (2) “real-time audio” interventions, (3) “asynchronous” interventions, and (4) “combined” interventions. To analyze changes in HbA_1c_, suitable RCTs were pooled and the average was determined. An HbA_1c_ decrease of –1.15% (95% CI –1.84% to –0.45%), yielding an HbA_1c_ value of 6.95% (SD 0.495), was shown in studies using 6-month “real-time video” interventions.

**Conclusions:**

Telemetric interventions clearly improve HbA_1c_ values in both the short term and the long term and contribute to the effective management of T2DM. More studies need to be done in greater detail.

## Introduction

Diabetes mellitus is a chronic burden, with a prevalence that is increasing worldwide [1]. In 2019, approximately 463 million adults were diagnosed with diabetes [1]. By 2045, the International Diabetes Federation (IDF) projects an increase of 51% up to approximately 700 million people diagnosed with diabetes [1]. The IDF also estimates that one-half of individuals living with diabetes are undiagnosed [1]. According to the American Diabetes Association, type 2 diabetes mellitus (T2DM) is the most prevalent type of diabetes and represents approximately 90% to 95% of all diabetes cases [2]. Common risk factors that appear to lead to T2DM are increasing age, increasing BMI, and lack of physical activity [2]. From a pathophysiological perspective, T2DM emerges mainly because of the progressive loss of beta-cell insulin secretion due to insulin resistance. Typically, however, relative insulin deficiency, as well as central and peripheral insulin resistance, arises [2].

T2DM is closely associated with diabetic microvascular complications—such as nephropathy, retinopathy, and neuropathy [3]—and macrovascular complications—such as coronary heart disease, stroke, and peripheral artery disease [4], as well as other comorbidities and general complications. In addition, cardiovascular disease is the main cause of death in patients with T2DM [4].

Therefore, optimal glycemic management is crucial [3]. Recent studies have reported positive effects of telemetric interventions on diabetes management [5,6]. Telemetry, defined as “a mode of delivering healthcare services through the use of telecommunications technologies, including but not limited to asynchronous and synchronous technology, and remote patient monitoring technology, by a healthcare practitioner to a patient or a practitioner at a different physical location than the healthcare practitioner” [7], may be a promising approach to improve the clinical effectiveness of T2DM management. This digital field of application is constantly evolving and expanding [8]. Telematics, the science of telecommunication and informatics, developed in the 1970s, and telemedicine emerged as a part of telematics in the 1970s and 1980s [8]. For a long time, the physical distance between the user groups was the dominant characteristic of telemedicine. The emergence of the internet in the 1990s opened up new communication channels. As a result, the focus was no longer on distance but on the fundamental application of technologies to overcome distance [8]. Electronic health (eHealth), characterized as health management based on electronic systems and communication, emerged from this idea [8]. The new concept of digital health combines the digital and genomic-proteomic revolutions with health care and everyday life [8].

In this systematic meta-review [9], we focused on telemetric communication pathways between health care professionals and patients. We aimed to update the evidence for and clinical effectiveness of telemetric approaches in the context of T2DM management considering different study designs such as randomized controlled trials (RCTs), clinical trials (CTs), systematic reviews (SRs), and meta-analyses (MAs). Furthermore, we focused on main clinical outcomes, such as glycated hemoglobin A_1c_ (HbA_1c_), blood pressure (BP), fasting blood glucose (FBG), BMI, diabetes-related quality of life (DRQoL), and health-related quality of life (HRQoL), as well as the cost-effectiveness, time savings, and clinical effectiveness of telemetric interventions in general. HbA_1c_ is one of the major clinical parameters in T2DM and therefore our main focus.

To our knowledge, this study is the first and only systematic meta-review of telemetric interventions in T2DM management with respect to the following special features: we developed and applied a unique classification system for analyzing telemetric interventions and provide detailed insights by including several study designs and a wide range of clinical outcomes.

### Research Design and Methods

#### Search Strategy

A systematic search was conducted targeting the period between January 2008 and April 2020. No protocol has been published. Keywords (diabetes mellitus, telemetry, telemonitoring, and telemedicine) were selected from the MEDLINE Medical Subject Headings and EMBASE Subject Headings databases and searched in titles/abstracts (Multimedia Appendix 1). In general, the steps were as follows: (1) search in five relevant databases, (2) eliminate duplicates, (3) screen titles and abstracts, (4) assess peer-reviewed publications for eligibility, (5) perform additive research via reference lists, (6) select T2DM studies, (7) extract relevant data, and (8) classify the publications.

#### Study Selection

Publications addressing telemetric interventions targeting T2DM management were included.

Telemetry was defined as “a mode of delivering healthcare services through the use of telecommunications technologies, including but not limited to asynchronous and synchronous technology, and remote patient monitoring technology, by a healthcare practitioner to a patient or a practitioner at a different physical location than the healthcare practitioner” [7]. We included video consultations, telephone counselling, asynchronous communication by email, SMS text messaging, internet/web-based platforms, and mixed forms.

Studies were screened and selected by two independent reviewers. Disagreements were resolved by a consensus-based discussion. We selected studies that met the following inclusion criteria: (1) peer-reviewed articles and studies; (2) written in English or German; (3) study design was an SR, MA, CT, or RCT; and (4) included interventions that involved direct interaction between patients and health care professionals through feedback and data transmission. We also considered quantitative and qualitative studies.

Smartphone/mobile app–based interventions were excluded and analyzed separately in another publication. We also rejected publications that observed mixed populations (eg, pooled patients with T1DM and T2DM), provided pooled data with other digital applications, addressed prevention or diagnosis, or focused on the presentation of technologies. Multimedia Appendix 2 shows the PRISMA (Preferred Reporting Items for Systematic Reviews and Meta-Analyses) flow diagram.

#### Data Extraction

Year of publication, location of the study, duration of the intervention, study design, sample sizes, intervention and control groups used, frequency of contact, feedback methods, outcomes, effects, statistical significance, and conclusions were extracted from each publication (step 7).

#### Study Classification and Analysis

**Figure 1:**
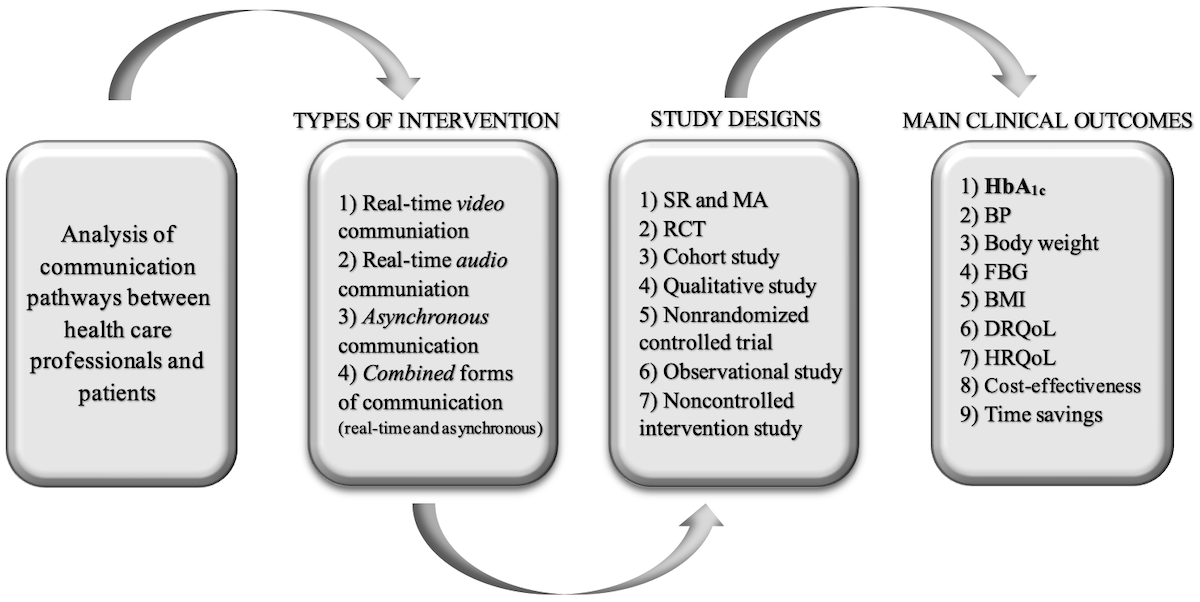
Study classification procedure. BP: blood pressure; DRQoL: disease-related quality of life; FBG: fasting blood glucose; HbA_1c_: glycated hemoglobin A1c; HRQoL: health-related quality of life; MA: meta-analysis; RCT: randomized controlled trial; SR: systematic review.

For analysis, the interventions were classified a priori (step 8) based on the technologies used, study design, and outcomes (Figure 1).

- “Real-time video” interventions (12 intervention studies): synchronous, face-to-face communication by videoconferencing and video consulting.
- “Real-time audio” interventions (17 intervention studies): synchronous communication by telephone calls (telephone coaching and counselling).
- “Asynchronous” interventions (28 intervention studies): asynchronous communication by email, SMS text messaging, internet/web-based platforms, server, home gateway, and post.
- “Combined” interventions (33 intervention studies): interventions involving real-time (ie, synchronous) and asynchronous communication, with a subgroup of “video clips” (interventions providing educational videos).

We conducted a small subgroup MA to assess whether the impact of the four intervention types, as well as the short- and long-term effects on the management of HbA_1c_ concentrations, differed. To determine the change in HbA_1c_, we pooled appropriate RCTs and calculated the differences in means and 95% CIs for the intervention and control groups at the study end points. RCTs in which the changes from baseline to the end of the study were reported as a percentage were included. Studies in which the control group received telemetric support were excluded. Mean deviations and SDs were extracted unchanged.

In addition, the publication bias was assessed visually as a funnel plot using HbA_1c_ values based on the RCTs and the mean differences (MDs) from our subgroup MA.

We also pooled the number of patients, specifically the number of unique patients as well as the number of patient cases related to the outcomes. In the former scenario, each patient occurred only once, addressing the number of individual patients (without SRs and MAs), and in the latter scenario, with a focus on specific outcomes, patient cases were analyzed based on the respective outcomes and thus may have been included several times (including SRs and MAs).

## Results

### Description of Studies

Our search strategy identified 1647 citations. After removing duplicates, 1116 studies were screened and 875 ineligible papers excluded. After assessing 241 studies with full text, 72 inappropriate studies were rejected. As an interim result, 189 studies were identified, of which 23 focused on type 1 diabetes mellitus, 99 focused on T2DM, 11 focused on gestational diabetes, and 51 focused on mixed populations. In this systematic meta-review, we included 99 suitable T2DM publications, analyzing 15,939 patients and 82,439 patient cases. A list of the included studies is provided in Multimedia Appendix 3.

Baseline characteristics of the studies are summarized in Table 1. Of the 99 studies, 10 were SRs and MAs, 73 were RCTs, 9 were qualitative examinations, 2 were cohort studies, 2 were non-RCTs, 2 were observational studies, and 1 was a noncontrolled intervention study. When classifying the studies according to location and type of intervention, SRs and MAs were excluded due to their heterogeneity, and thus 89 studies were taken into account. Of these 89 studies, 35 were done in the United States, 21 in Asia, 20 in Europe, 6 in Australia, 3 in Canada, 2 in Brazil, and 2 in Turkey.

In total, 12 “real-time video,” 17 “real-time audio,” 28 “asynchronous,” 33 “combined,” and 3 “video clip” interventions were classified. One study matched the classification criteria for two categories [10]. A detailed summary of all studies is shown in Multimedia Appendix 4.

A descriptive examination of the funnel plot created using HbA_1c_ values indicated a mild form of asymmetry (Multimedia Appendix 8).

**Table 1:** Baseline characteristics of reviewed studies.

Studies				n (%)
**All studies (N=99)**				
	**Study design**			
		SRs^a^ and MAs^b^		10 (10)
		**Randomized controlled trials (total)**		73 (74)
			Pilot studies	3 (3)
		Cohort studies		2 (2)
		Qualitative studies		9 (9)
		Nonrandomized controlled trials		2 (2)
		Observational studies		2 (2)
		Noncontrolled intervention studies		1 (1)
	**Years**			
		2008-2011		21 (21)
		2012-2014		26 (26)
		2015-2017		32 (32)
		2018-2020		19 (19)
**All studies, excluding SRs and MAs (n=89)**				
	**Location**			
		United States		35 (39)
		Canada		3 (3)
		Brazil		2 (2)
		Europe		20 (23)
		Asia		21 (24)
		Australia		6 (7)
		Turkey		2 (2)
	**Intervention**			
		Real-time video		12 (14)
		Real-time audio		17^c^ (19)
		Asynchronous		28^c^ (32)
		**Combined forms (total)**		33 (37)
			“Video clips” subgroup	3 (3)

^a^SRs: systematic reviews.

^b^MAs: meta-analyses.

^c^One study matched the criteria for two categories.

### Impact on Main Outcomes

An overview of significant and not significant intervention effects on HbA_1c_, BP, FBG, BMI, DRQoL, HRQoL, cost-effectiveness, time savings, and clinical effectiveness is displayed in Multimedia Appendix 5. Multimedia Appendix 6 shows the significant effects on the main outcomes. Briefly, 85% (84/99) of the intervention studies found explicit beneficial effects due to telemetric interventions, depending on the outcomes studied (see Multimedia Appendix 4).

### SRs and MAs (n=10)

#### HbA_1c_ (n=8)

All SRs and MAs reported clear decreases in HbA_1c_ values (*P<.*05) by implementing telemetric interventions [5,11-17]. MAs (5/5, 100%) indicated that telemetry was significantly associated with an obvious improvement between –0.37% and –0.55% in HbA_1c_ values compared with the usual care (*P<.*001 [15], *P<.*001 [13], *P<.*001 [12], *P<.*001 [5], and *P<.*05 [10]).

#### BMI (n=1)

According to Kim et al [5], telemonitoring was associated with a significantly reduced BMI (weighted MD=–0.25 kg/m², 95% CI –0.49 to –0.01, *I*²=16.7%) compared with usual care.

#### Cost-Effectiveness (n=1)

Due to the heterogeneous data situation, Zhai et al [13] could not draw any conclusions regarding cost-effectiveness.

### “Real-Time Video” Interventions (n=12)

#### HbA_1c_ (n=9)

Overall, 89% (8/9) of the studies reported a clear reduction in HbA_1c_ values. More specifically, five RCTs (5/9, 56%) indicated significant positive effects (*P=.*022 [18], *P=.*004 [19], *P=.*023 [20], *P<*.05 [21], and *P=*.013 [22]). For example, HbA_1c_ values declined significantly in an intervention group with weekly video conferences compared with a control group (0.49% versus 0.17%; *P=*.013) [22].

#### FBG (n=3)

Overall, definite improvements regarding FBG were documented. Tavsanli et al [22] (weekly video conferences) and Rasmussen et al [20] (average 4.1 video consultations in 6 months) reported clearly lower FBG levels in the intervention group compared with control groups (*P>.*05 [22] and *P<.*015 [20]), whereas Hansen et al [18] provided monthly video conferences additional to usual care and reported no substantial changes in FBG levels in relation to the study (significance not reported).

#### BP (n=3)

In general, most RCTs observed no essential changes in systolic and diastolic BP measurements (intergroup *P>.*05 [20] and significance not reported [18]). However, a clear improvement in BP was seen in a 12-month videoconferencing intervention, as reported by Davis et al [19], although the effect was not significant compared with a control group (intervention systolic BP 130.8 mmHg, SD 3.6 mmHg, versus 127.6 mmHg, SD 4.0 mmHg, *P=.*76; diastolic BP 72.7 mmHg, SD 2.1 mmHg, versus 70.2 mmHg, SD 2.2 mmHg, *P=.*64).

#### Body Weight (n=1)

Rasmussen et al [20] showed a significantly higher weight loss with in-person clinic visits (–1.7 kg) compared with video consultations (–0.6 kg; *P=.*023).

#### BMI (n=2)

Hansen et al [18] found no obvious changes in terms of BMI, whereas Davis et al [19] indicated substantial improvements compared to usual care, although the finding was not significant (30.6 kg/m², SD 1.4 kg/m² versus 35.8 kg/m², SD 1.4 kg/m²; *P=.*73).

#### HRQoL (n=1)

Hansen et al [18] noted that significant changes in mental or physical health rankings were not detected.

#### Time Savings (n=1)

Gordon et al [23] revealed shorter travel times and less time in waiting rooms according to interviews with participants, although no statistical measurements were performed.

#### Enablers and Barriers (n=2)

Carlisle and Warren [24] suggested that consumer-friendly technologies and the integration of telemetry into everyday lives are important for the successful implementation of telemetry interventions.

### “Real-Time Audio” Interventions (n=17)

#### HbA_1c_ (n=9)

In summary, all studies showed precise improvements in HbA_1c_ levels with audio interventions in real time. Odnoletkova et al [25] and Walker et al [26] reported significant improvements in their intervention groups compared with matched control groups (intervention group: –0.2%, 95% CI –0.3 to –0.1, *P=.*003 [25]; and intervention group versus control group: –0.23% versus 0.13%, *P=.*04 [26]). Sarayani et al [27], Trief et al [28], Maslakpak et al [29], Blackberry et al [30], Benson et al [31], and Vasconcelos et al [32] displayed clear, but not significant, improvements in HbA_1c_ levels compared with control groups (*P>.*05). Notably, some control groups [27-29,31] received some forms of telemetric or even educational support. Interestingly, Walker et al [26] found that patients who completed at least six phone calls with a health educator over a 12-month period had significant reductions in their HbA_1c_ concentrations (*P<.*05).

The RCT of McMahon et al [10] was classified in two categories (“real-time audio” and “asynchronous” interventions) because it involved the comparison of a telephone-based intervention with two “asynchronous” interventions. HbA_1c_ decreased at a rate of 0.32% every 3 months for the online arm, 0.36% for the telephone arm, and 0.41% for the web training arm (all *P<.*001).

#### FBG (n=2)

The “real-time audio” intervention studies showed obvious improvements in FBG. In the study by Varney et al [33], FBG levels clearly improved in subjects in the intervention group (8.9 mmol/L, 95% CI 8.0 to 9.7, to 8.5 mmol/L, 95% CI 7.7 to 9.4) compared with those in the control group (*P=.*02), but not in a long-term way, while Maslakpak et al [29] outlined distinct, but not significant, differences between telephone and control groups (*P=.*766).

#### BP (n=3)

In general, all “real-time audio” intervention studies reported clear improvements in BP. Trief et al [28] showed a greater improvement in systolic BP in the “individual calls” group than in the “diabetes education” group at 8 months (*P=.*021). Vasconcelos et al [32] reported improvements in systolic BP (130.25 mmHg to 125.87 mmHg; *P=.*171) and diastolic BP (72.12 mmHg to 71.12 mmHg; *P=.*640). In addition, Varney et al [33] indicated significant improvements in diastolic BP within the telephone group (80 mmHg, 95% CI 76 to 84, to 74 mmHg, 95% CI 71 to 77), but these were not sustained.

#### Body Weight (n=1)

According to Odnoletkova et al [25], the difference between the groups in favor of telecoaching was a change in body weight of –1.1 kg (*P=.*004).

#### BMI (n=3)

In general, all trials noted slight improvements in BMI. Odnoletkova et al [25] and Trief et al [28] reported significant improvements between groups (*P=.*003 [25] and *P=.*021 [28]), whereas Vasconcelos et al [32] indicated a slight decrease (29.99 kg/m² to 29.96 kg/m²) that was not significant (*P=.*764).

#### Cost-Effectiveness (n=2)

“Real-time audio” interventions appear to be moderate in terms of cost-effectiveness (no statistical significances reported). Schechter et al [34] concluded that the costs were moderate relative to the benefits, whereas Varney et al [35] revealed that the cost of a 10-year intervention was covered by the financial savings, with a tendency for health profits.

### “Asynchronous” Interventions (n=28)

#### HbA_1c_ (n=24)

Overall, the majority of the studies (all RCTs; 23/34, 96%) reported apparent improvements. Eleven RCTs reported significant improvements in HbA_1c_ in the intervention groups compared with the control groups (*P<.*05) [36-46], whereas 3 RCTs showed significant beneficial effects within their intervention groups (*P<.*05) [47-49]. In addition, 5 RCTs found improvements in the intervention groups compared with matched controls, but the results were not statistically significant (*P>.*05) [50-54]. Ramadas et al [55], Tildesley et al [38], and Cho et al [56] mentioned significant improvements within their intervention groups (*P=.*004 [55]; real-time continuous glucose monitoring, *P<.*001, versus internet blood glucose monitoring system, *P<.*05 [38]; and *P<.*01 [56]), but the differences between groups were not significant (*P>.*05).

#### FBG (n=2)

The studies showed significant improvements in FBG levels in the intervention groups compared with control groups (8.9 mmol/L, SD 3.9 mmol/L, versus 7.9 mmol/L, SD 2.5 mmol/L, *P=.*015 [55]; and *P=.*005 [46]).

#### BP (n=3)

In summary, the publications reported apparent improvements in BP. Wild et al [39] found significant improvements in systolic BP (*P=.*017) and diastolic BP (*P=.*006) in the intervention group compared with the control group. Wakefield et al [40] also found a significant decrease in systolic BP in the high-intensity arm of the intervention (home telehealth device with algorithm; *P=.*01). Fang and Deng [44] found improvements in systolic BP (*P=.*069) and diastolic BP (*P=.*693) in the treatment group, but they were not significant.

#### Body Weight (n=3) and BMI (n=2)

In general, the studies revealed clear beneficial effects of “asynchronous” interventions on both body weight and BMI. For example, Luley et al [41] showed large significant improvements in body weight (–11.8 kg, SD 8.0 kg; both inter- and intragroup comparisons with *P=.*000) and BMI (–4.1 kg/m²; both intergroup and intragroup comparisons with *P=.*00).

#### HRQoL (n=1)

No clinically important improvements in HRQoL were seen according to Dario et al [51].

#### Cost-Effectiveness (n=1)

A weight-loss telemonitoring intervention from Luley et al 2011 [41] showed an effective decline in medication costs of €83 (US $101) per patient in 6 months.

#### Time Savings (n=1)

Cho et al [57] showed a significant time savings for physicians of approximately 55% focusing on patients with HbA_1c_ levels greater than 6.5% (*P<.*05).

### “Combined” Interventions (n=33)

#### HbA_1c_ (n=24)

In general, most publications (21/24, 88%) reported clear significant improvements in HbA_1c_ (*P<.*05). Three of these studies were RCTs that achieved significant improvements within their intervention groups (*P<.*001 [58]; *P=.*27 [59]; and P value not reported [60]) but not significant differences between the intervention and control groups (*P>.*05).

#### FBG (n=6)

Overall, the studies showed mostly positive effects of “combined” interventions on FBG [58,61-64]. For example, Zhou et al [64] and Jeong et al [58] found significant reductions in FBG levels compared with the control groups (8.73 mmol/L to 7.06 mmol/L, *P<.*001 [64]; and –12.28 mg/dL, SD 41.20 mg/dL, *P=.*027 (telemedicine group [58]).

#### BP (n=13)

Approximately 85% (11/13) of the “combined” intervention studies reported beneficial effects on BP [59-61,63-70]. Kempf et al [70] and Crowley et al [65] found significant improvements compared with control groups (systolic BP, *P<.*01 [70]; and systolic BP, *P=.*035, and diastolic BP, *P=.*013 [65]). However, some RCTs noted improvements in their intervention groups (ie, *P<.*05) but no significant differences between the groups (*P>.*05) [60,66-69,71]. Additionally, Kesavadev et al (cohort study with 1000 participants [63]) and Dienstl et al (observational study [61]) indicated similar significant improvements in systolic and diastolic BP (*P<.*01 [63] and *P<.*001 [61]).

#### Body Weight (n=7)

Most “combination” intervention studies (5/7, 71%) found clear improvements in body weight [60,61,69,70,72]. For example, Kempf et al [70] reported a significant reduction of 6.2 (SD 4.6) kg in the intervention group compared with the control group (–1.0 kg, SD 3.4 kg; *P<.*01).

#### BMI (n=9)

The majority of publications (7/9, 78%) showed an apparent reduction of BMI. Significant improvements were outlined by 6 studies (intragroup *P=.*047 [59], intragroup *P<.*01 [63], intragroup *P<.*001 [61], intergroup *P=.*036 [73], intergroup *P<.*01 [70], and intergroup *P<.*05 [74]). For example, Kesavadev et al [63] (n=1000 patients) showed a significant reduction of 0.3 kg/m² (*P<.*01) and Kempf et al [70] reported –2.1 (SD 1.5) kg/m² in the intervention group versus –0.3 (SD 1.1) kg/m² in the control group (*P<.*01).

#### DRQoL (n=3) and HRQoL (n=1)

All studies found clear improvements in DRQoL and HRQoL. Kempf et al [70] and Nicolucci et al [69] showed significant intergroup improvements in HRQoL (*P<.*01 and *P<.*03, respectively). Jha et al [62] and Dienstl et al [61] (observational studies) reported significant beneficial effects with regard to DRQoL (*P=.*015 and *P<.*001, respectively).

#### Cost-Effectiveness (n=2)

Warren et al [75] and Kesavadev et al [63] reported that “combined” interventions are cost-effective. The total costs for the internet-based treatment group were lower than those for the control group (mean US $3781 versus US $4662; *P<.*001 [75]). According to Kesavadev et al [63], the extra cost was US $9.66/month (significance not reported), but money and time saved in physical visits made up for the extra costs.

#### Time Savings (n=1)

Hsu et al [72] reported great time savings with a cloud-based diabetes management program compared with standard face-to-face care (22.5-minute versus 68.8-minute visit time; significance not reported).

### “Combined” Interventions—“Video Clips” Subgroup (n=3)

#### HbA_1c_ (n=3)

All studies reported significant reductions in HbA_1c_ compared with control subjects (*P<.*001 [76], *P=.*005 [77], and *P=.*013 [78]).

#### BP and Body Weight (n=1)

Tang et al [76] detected improvements in BP (systolic BP, *P=.*306, and diastolic BP, *P=.*374) but no effects on body weight (*P=.*232).

### Short- and Long-Term Effects on HbA_1c_ Values (n=41)

Short- and long-term effects based on the comparison of HbA_1c_ values between the intervention and control groups at the study end points were investigated. Patients’ changes in HbA_1c_ from baseline to the end of the study of 41 RCTs are presented in Multimedia Appendix 7.

#### “Real-Time Video” Interventions

A small MA showed that, compared with the control group, 6-month interventions (n=2) were associated with a greater effect size (MD=–1.15%, 95% CI –1.84 to –0.45) than 12-month interventions (n=2) (MD=–0.6%, 95% CI –0.99 to –0.21).

#### “Real-Time Audio” Interventions

The subgroup analysis revealed an effect size, compared to usual care, of MD=–0.37% (95% CI –0.79 to 0.05) for 6-month interventions (n=3) compared with –0.5% in the 3-month intervention [29] and –0.06% in the 18-month intervention [30].

#### “Asynchronous” Interventions

The greatest effect was seen in 12-month interventions (n=2) (MD=–0.77%, 95% CI –2.25 to 0.72), followed by 6-month interventions (n=8) (MD=–0.57%, 95% CI –0.75 to –0.39), and 3-month interventions (n=3) (MD=–0.38%, 95% CI –0.54 to –0.22).

#### “Combined” Interventions

The 3-month interventions (n=5) had the greatest effect (MD=–0.65%, 95% CI –0.98 to –0.31), whereas 6-month interventions (n=7) had a slightly smaller effect (MD=–0.50%, 95% CI –0.71 to –0.30). In comparison, the effect of 12-month interventions (n=4) was even smaller (MD=–0.25%, 95% CI –0.73 to 0.24). The subgroup “video clip” interventions (n=2) showed a reduction of MD=–0.23 (95% CI –0.23 to –0.23).

## Discussion

### Principal Results

Telemetry is a viable alternative to usual care for patients with T2DM and can lead to improvements in a wide range of outcomes. The inclusion of evidence from different study designs, such as reviews and trials, in our review strengthens the conclusion that use of telemetric interventions can be feasible in a clinical setting. Other reviews have also recently presented an improvement of clinical outcomes through telemetry and especially a trend toward a reduction in HbA_1c_ levels [16,79]. Our results suggest that telemetry generated clinically meaningful reductions in HbA_1c_ levels. Telemetry has the advantage of helping people who are restricted due to geographic location or a lack of resources [14]. In the time of COVID-19 in particular, the advantages and potential of remote diabetes management becomes even more important.

### Impact of Telemetric Interventions on HbA1c

In general, all types of telemetric interventions clearly improved HbA_1c_. All SRs and MAs also clearly showed that telemetric interventions improve HbA_1c_ specifically, as well as the management of T2DM generally. Furthermore, “real-time video” interventions with a duration of 6 months were the most effective in reducing HbA_1c_. These interventions showed clear improvements in HbA_1c_ levels in patients diagnosed with T2DM compared to usual care (MD=–1.15%, 95% CI –1.84 to –0.45). Overall, the effects in the subgroup analysis in terms of the improvement of HbA_1c_ values had MDs between –1.15% and –0.25%. These obvious decreases in HbA_1c_ may indicate a novel and additional approach to diabetes care since these therapeutic effects could be accomplished by telemetric intervention alone. However, to optimize glucose homeostasis, individual telemetric approaches may be considered in terms of individual diabetes care as an addition to established therapeutic approaches [4].

### Impact of Telemetric Interventions on Main Clinical Outcomes

Through the use of “combined” interventions, FBG levels improved effectively, which was shown by a moderate number of studies. With “asynchronous” and “real-time audio” interventions, few studies showed an improvement in FBG values. However, the data were inconsistent as to whether “real-time video” interventions reduce FBG effectively.

BP measurements decreased by applying “combined” interventions in a moderate number of the reviewed studies. “Asynchronous” and “real-time audio” interventions also improved BP, but there were comparatively few examinations. Moreover, the study situation for “real-time video” interventions was found to be rather inconsistent.

Body weight decreased in a moderate number of studies by using “combined” interventions effectively. “Real-time audio” interventions also clearly reduced body weight in a few investigations. However, the study situation for “real-time video” interventions was not consistent.

BMI decreased effectively in several studies by using “combined” interventions. The few studies available indicated that “asynchronous” and “real-time audio” interventions decreased BMI. In contrast, the study situation for “real time video” was inconsistent.

Only “combined” interventions showed effective improvements regarding DRQoL and HRQoL, but there were few studies that examined DRQoL and very few studies of HRQoL compared with the other clinical outcomes. “Real-time audio,” “asynchronous,” and “combined” interventions were potentially cost-effective, but there was only a small number of studies. In addition, “real-time video,” “asynchronous,” and “combined” interventions occasionally showed time savings, although again, few studies examined these outcomes.

### Impact and Comparison of Different Telemetric Interventions

“Real-time video” interventions did improve HbA_1c_ clearly and effectively in short-term and long-term ways in a large number of studies. Weekly videoconferencing seems to be very effective in terms of reducing HbA_1c_. Due to the heterogeneity of the studies, the results regarding FBG, BP, body weight, BMI, and QoL may be rather inconsistent. However, they all have in common that user-friendly technologies were considered in the development of the interventions and that telemetry was anchored in people’s everyday lives, both of which are necessary for optimal results.

“Real-time audio” interventions proved to be effective in reducing HbA_1c_, as demonstrated in numerous studies. Some studies indicated that there is also a clear beneficial impact of these interventions on FBG, BP, body weight, and BMI. Additionally, “real-time audio” interventions were shown to be cost-effective by the limited studies available.

Furthermore, a large number of studies pointed out that “asynchronous” interventions improved HbA_1c_ effectively. These interventions also improved FBG, BP, and BMI, and showed very positive results in terms of cost-effectiveness and time savings, but few studies using “asynchronous” interventions were available for review.

Most studies assessed “combined” interventions (real-time and asychronous communication). Numerous studies indicated that “combined” interventions improved HbA_1c_ values effectively. Furthermore, a moderate number of interventions had a favorable impact on FBG, BP, BMI, and body weight. In terms of DRQoL and HRQoL, there were few studies to examine these outcomes, but the available studies showed positive tendencies. Additionally, cost-effectiveness and time savings of telemetric interventions showed a positive trend, but sufficient data were lacking.

From our point of view, telemetric T2DM management enhances patient compliance, enables intensive monitoring, and empowers patients to deal with and understand their disease. For a successful implementation of telemetric approaches, it is also essential that the technology is user-friendly, that telemetric T2DM management can be easily integrated into everyday life, and that it is tailored to the patient and his or her life circumstances [24].

Furthermore, we would like to point out that telemetric interventions differ not only in terms of their technologies but also in terms of their contextual focus (eg, nutrition, exercise, etc) and that this aspect should be taken into account when interpreting the results.

### Study Limitations

Although the exclusion criteria were observed, the included studies displayed a wide variation in terms of study design, technical and interventional approaches, duration, and frequency of contact with health care providers (in both the intervention and the control groups), as well as sample size and statistical evaluations used. Due to this heterogeneity, as well as to the small size of our MA, there may be potential for bias. For the same reasons, methodological quality and statistical evaluations could not be carried out. Some studies achieved improvements that were significant within the intervention groups but not between the groups, and methodological weaknesses may have been responsible for that.

### Comparison With Prior Work

Other research groups have displayed similar results. Numerous other SRs and MAs, which were included in this review, reported significant decreases in HbA_1c_ values (*P<.*05) from the implementation of telemetric interventions [5,11-17]. Su et al [79] examined 55 RCTs and concluded that telemedicine effectively improved clinical outcomes as well as T2DM management compared with usual care. Lee et al [16], who included 4 SRs reporting on 29 studies, concluded that telemetry was a very effective therapeutic approach in terms of decreasing HbA_1c_. According to a review and network MA by Lee et al [14], over a 6-month follow-up, telemedicine reduced HbA_1c_ by a mean of 0.43% (95% CI −0.64% to −0.21%). The authors concluded that all telemedical strategies, with the exceptions of telecase management and telementoring, were effective in reducing HbA_1c_ in a clinically meaningful way. Furthermore, Mushcab et al [17] showed that telemonitoring effectively improved HbA_1c_ levels and quality of life. They also observed a high acceptance of web-based systems.

Our research builds on these previous findings, incorporating a large number of studies (n=99), patients (n=15,939), and patient cases (n=82,436) and considering a range of main clinical outcomes in terms of T2DM management. Interestingly, there may be differences in telemetric approaches in terms of T2DM versus type 1 diabetes mellitus management [79], but these still need to be analyzed in more detail.

### Conclusions

To our knowledge, this is the first systematic meta-review analyzing telemetric approaches in T2DM management, including a wide range of important clinical outcomes and technologies.

Viewed together, telemetric interventions clearly improve HbA_1c_ values in the short term and long term specifically and T2DM care generally. Moreover, “real-time video” interventions with a duration of 6 months showed the greatest effect in terms of improving HbA_1c_ values in a sustained way. “Combined” interventions (real-time and asynchronous communication) appeared to be most effective in improving FBG, BP, body weight, BMI, and quality of life.

In conclusion, telemetric interventions clearly improve HbA_1c_ and T2DM management effectively. More studies need to be done, especially with a focus on main clinical outcomes.

## Appendix

Multimedia Appendix 1 Database search strings.

Multimedia Appendix 2 Systematic meta-review search and selection procedure based on PRISMA (Preferred Reporting Items for Systematic Reviews and Meta-Analyses) guidelines.

Multimedia Appendix 3 List of included studies.

Multimedia Appendix 4 Summary of the studies included in this systematic meta-review.

Multimedia Appendix 5 Impact on main clinical outcomes, significant and not significant effects.

Multimedia Appendix 6 Significant effects on main clinical outcomes.

Multimedia Appendix 7 Changes in glycated hemoglobin (HbA1c) values (%) in intervention and control groups from baseline to end of the study (n=41 randomized controlled trials).

Multimedia Appendix 8 Funnel plot using glycated hemoglobin (HbA1c) based on the randomized controlled trials from the subgroup meta-analysis.

## Abbreviations

BP: blood pressure
CT: clinical trial
DRQoL: diabetes-related quality of life
FBG: fasting blood glucose
HbA1c: glycated hemoglobin A1c
HRQoL: health-related quality of life
IDF: International Diabetes Federation
MA: meta-analysis
MD: mean difference
PRISMA: Preferred Reporting Items for Systematic Reviews and Meta-Analyses
RCT: randomized controlled trial
SR: systematic review
T2DM: type 2 diabetes mellitus
