# Supplementary material for: Effect of Telemetric Interventions on Glycated Hemoglobin A1c and Management of Type 2 Diabetes Mellitus: Systematic Meta-Review
Source: J Med Internet Res. 2021 Feb 17;23(2):e23252. doi: 10.2196/23252 (PMC7929744; doi:10.2196/23252)
Supplement: Multimedia Appendix 1 [file jmir_v23i2e23252_app1.pdf]

Database search strings.

	PUBMED	EMBASE	COCHRANE	CINAHL	WEB OF SCIENCE CORE COLLECTION
**Keywords**	diabetes mellitus, gestational diabetes, telemetry, telemedicine, telemonitoring	diabetes mellitus, pregnancy diabetes mellitus, gestational diabetes, telemetry, telemedicine, telemonitoring	diabetes mellitus, gestational diabetes, telemetry, telemedicine, telemonitoring	diabetes mellitus, gestational diabetes, telemetry, telemedicine, telemonitoring	diabetes mellitus, gestational diabetes, telemetry, telemedicine, telemonitoring
**Filters**	clinical trial, meta-analysis, randomized controlled trial, systematic review; publication date from 2008/01/01 to 2020/12/31; English; German	clinical trial, meta-analysis, randomized controlled trial, systematic review; 2008-2020; English; German	cochrane reviews, trials and clinical answers; from January 2008 to April 2020; English; German	clinical trial, meta-analysis, randomized controlled trial, systematic review; 2008/01/01-2020/04/02; English, German	2008-2020; English; German
**Search**	((((("diabetes mellitus"[Title/Abstract]) OR "Diabetes Mellitus"[MeSH Terms]) OR "gestational diabetes"[Title/Abstract]) OR diabetes, gestational[MeSH Terms])) AND ((((("Telemetry"[Mesh]) OR "Telemedicine"[Mesh]) OR telemonitoring[Title/Abstract]) OR "telemetry"[Title/Abstract]) OR "telemedicine"[Title/Abstract]))	((‘diabetes mellitus’/exp OR ‘pregnancy diabetes mellitus’/exp OR ‘diabetes mellitus’:ab,ti OR ‘gestational diabetes’: ab,ti) AND (‘telemetry’/exp OR ‘telemedicine’/exp OR ‘telemonitoring/exp OR ‘telemedicine:ab,ti OR ‘telemonitoring:ab,ti OR ‘telemetry’:ab,ti))	((MeSH [Diabetes Mellitus] OR MeSH [Diabetes, Gestational] OR “diabetes mellitus”:ti,ab OR “gestatinal diabetes”:ti,ab) AND (MeSH [Telemedicine] OR MeSH [Telemetry] OR “telemedicine”:ti,ab OR “telemetry”:ti,ab OR “telemonitoring”:ti,ab))	((MH “diabetes mellitus” OR TI “diabetes mellitus” OR AB “diabetes mellitus” OR TI “gestational diabetes” OR AB “gestational diabetes”) AND (MH telemedicine OR MH telemetry OR TI telemetry OR AB telemetry OR TI telemedicine OR AB telemedicine OR TI telemoritoring OR AB telemonitoring))	((TOPIC “diabetes mellitus” OR TOPIC “gestational diabetes”) AND (TOPIC telemetry OR TOPIC telemedicine OR TOPIC telemonitoring))
