# Supplementary material for: Effect of Telemetric Interventions on Glycated Hemoglobin A1c and Management of Type 2 Diabetes Mellitus: Systematic Meta-Review
Source: J Med Internet Res. 2021 Feb 17;23(2):e23252. doi: 10.2196/23252 (PMC7929744; doi:10.2196/23252)
Supplement: Multimedia Appendix 3 [file jmir_v23i2e23252_app3.pdf]

List of included studies.

Andrews S.M., Sperber N.R., Gierisch J.M., Danus S., Macy S.L., Bosworth H.B., Edelman D., Crowley M.J. Patient perceptions of a comprehensive telemedicine intervention to address persistent poorly controlled diabetes. Patient Preference and Adherence 2017;11:469-478. PMID:28424543

Arora S, Peters AL, Burner E, Lam CN, Menchine M. Trial to examine text message-based mHealth in emergency department patients with diabetes (TExT-MED): a randomized controlled trial. Ann Emerg Med 2014;63(6):745-54.e6. PMID:24225332

Avdal EU, Kizilci S, Demirel N. The effects of web-based diabetes education on diabetes care results: a randomized control study. Comput Inform Nurs 2011;29(2):101-106. PMID:21099675

Barton AB, Okorodudu DE, Bosworth HB, Crowley MJ. Clinical Inertia in a Randomized Trial of Telemedicine-Based Chronic Disease Management: Lessons Learned. Telemed J E Health 2018;24(10):742-748. PMID:29341850

Benson GA, Sidebottom A, Hayes J, Miedema MD, Boucher J, Vacquier M, Sillah A, Gamam S, VanWormer JJ. Impact of ENHANCED (diEtitiaNs Helping pAtieNts CarE for Diabetes) Telemedicine Randomized Controlled Trial on Diabetes Optimal Care Outcomes in Patients with Type 2 Diabetes. J Acad Nutr Diet 2019;119(4):585-598. PMID:30711463

Bentley CL, Otesile O, Bacigalupo R, Elliott J, Noble H, Hawley MS, Williams EA, Cudd P. Feasibility study of portable technology for weight loss and HbA1c control in type 2 diabetes. BMC Med Inform Decis Mak 2016;16(92):1-15. PMID:27418275

Blackberry I.D., Furler J.S., Best J.D., Chondros P., Vale M., Walker C., Dunning T., Segal L., Dunbar J., Audehm R., Liew D., Young D. Effectiveness of general practice based, practice nurse led telephone coaching on glycaemic control of type 2 diabetes: The Patient Engagement and Coaching for Health (PEACH) pragmatic cluster randomised controlled trial. BMJ 2013;347(f5272). doi:10.1136/bmj.f5272

Bujnowska-Fedak MM, Puchala E, Steciwko A. The impact of telehome care on health status and quality of life among patients with diabetes in a primary care setting in Poland. Telemed J E Health 2011;17(3):153-163. PMID:21375410

Burner E, Lam CN, DeRoss R, Kagawa-Singer M, Menchine M, Arora S. Using Mobile Health to Improve Social Support for Low-Income Latino Patients with Diabetes: A Mixed-Methods Analysis of the Feasibility Trial of TExT-MED + FANS. Diabetes Technol Ther 2018;20(1):39-48. PMID:29227155

Carlisle K, Warren R. A qualitative case study of telehealth for in-home monitoring to support the management of type 2 diabetes. J Telemed Telecare 2013;19(7):372-375. PMID:24218347

Carter EL, Nunlee-Bland G, Callender C. A patient-centric, provider-assisted diabetes telehealth self-management intervention for urban minorities. Perspect Health Inf Manag 2011;8:1b. PMID:21307985

Cassimatis M, Kavanagh DJ. Effects of type 2 diabetes behavioural telehealth interventions on glycaemic control and adherence: a systematic review. J Telemed Telecare 2012;18(8):447-450. PMID:23209266

Castelnuovo G, Manzoni GM, Cuzziol P, Cesa GL, Tuzzi C, Villa V, Liuzzi A, Petroni ML, Molinari E. TECNOB: study design of a randomized controlled trial of a multidisciplinary telecare intervention for obese patients with type-2 diabetes. BMC Public Health 2010;10:204. PMID:20416042

Chen S-Y, Chang Y-H, Hsu H-C, Lee Y-J, Hung Y-J, Hsieh C-H. One-Year Efficacy and Safety of the Telehealth System in Poorly Controlled Type 2 Diabetic Patients Receiving Insulin Therapy. Telemed J E Health 2011;17(9):683-687. PMID:21882998

Cho JH, Choi YH, Kim HS, Lee JH, Yoon KH. Effectiveness and safety of a glucose data-filtering system with automatic response software to reduce the physician workload in managing type 2 diabetes. J Telemed Telecare 2011;17(5):257-262. PMID:21628421

Cho JH, Kim H-S, Yoo SH, Jung CH, Lee WJ, Park CY, Yang HK, Park JY, Park SW, Yoon KH. An Internet-based health gateway device for interactive communication and automatic data uploading: Clinical efficacy for type 2 diabetes in a multi-centre trial. J Telemed Telecare 2017;23(6):595-604. PMID:27381040

Cho JH, Kwon HS, Kim HS, Oh JA, Yoon KH. Effects on diabetes management of a health-care provider mediated, remote coaching system via a PDA-type glucometer and the Internet. J Telemed Telecare 2011;17(7):365-370. PMID:21933896

Cho JH, Lee HC, Lim DJ, Kwon HS, Yoon KH. Mobile communication using a mobile phone with a glucometer for glucose control in Type 2 patients with diabetes: as effective as an Internet-based glucose monitoring system. J Telemed Telecare 2009;15(2):77-82. PMID:19246607

Ciemins E, Coon P, Peck R, Holloway B, Min S-J. Using telehealth to provide diabetes care to patients in rural Montana: findings from the promoting realistic individual self-management program. Telemed J E Health 2011;17(8):596-602. PMID:21859347

Crowley MJ, Edelman D, McAndrew AT, Kistler S, Danus S, Webb JA, Zanga J, Sanders LL, Coffman CJ, Jackson GL, Bosworth HB. Practical Telemedicine for Veterans with Persistently Poor Diabetes Control: A Randomized Pilot Trial. Telemed J E Health 2016;22(5):376-384. PMID:26540163

Dario C, Toffanin R, Calcaterra F, Saccavini C, Stafylas P, Mancin S, Vio E. Telemonitoring of Type 2 Diabetes Mellitus in Italy. Telemed J E Health 2017;23(2):143-152. PMID:27379995

Davis RM, Hitch AD, Salaam MM, Herman WH, Zimmer-Galler IE, Mayer-Davis EJ. TeleHealth improves diabetes self-management in an underserved community: diabetes TeleCare. Diabetes Care 2010;33(8):1712-1717. PMID:20484125

Del Prato S, Nicolucci A, Lovagnini-Scher AC, Turco S, Leotta S, Vespasiani G. Telecare Provides comparable efficacy to conventional self-monitored blood glucose in patients with type 2 diabetes titrating one injection of insulin glulisine-the ELEONOR study. Diabetes Technol Ther 2012;14(2):175-182. PMID:22013886

Dienstl M, Kempf K, Schulz C, Kruse J, Martin S. Effect of Telemedicine on Glucometabolic Control and Quality of Life in Patients with Type 2 Diabetes Mellitus. Diabetologie Und Stoffwechsel 2011;6(3):164-169. doi:10.1055/s-0031-1271460

Dy P, Morin PC, Weinstock RS. Use of telemedicine to improve glycemic management in a skilled nursing facility: a pilot study. Telemed J E Health 2013;19(8):643-645. PMID:23758078

Egede LE, Walker RJ, Payne EH, Knapp RG, Acierno R, Frueh BC. Effect of psychotherapy for depression via home telehealth on glycemic control in adults with type 2 diabetes: Subgroup analysis of a randomized clinical trial. J Telemed Telecare 2018;24(9):596-602. PMID:28945160

Egede LE, Williams JS, Voronca DC, Knapp RG, Fernandes JK. Randomized Controlled Trial of Technology-Assisted Case Management in Low Income Adults with Type 2 Diabetes. Diabetes Technol Ther 2017;19(8):476-482. PMID:28581821

Fang R, Deng X. Electronic messaging intervention for management of cardiovascular risk factors in type 2 diabetes mellitus: A randomised controlled trial. J Clin Nurs 2018;27(3-4):612-620. PMID:28700102

Fernandes BSM, Reis IA, Torres HdC. Evaluation of the telephone intervention in the promotion of diabetes self-care: a randomized clinical trial. Rev Lat Am Enfermagem 2016;24:e2719. PMID:27579926

Fortmann AL, Gallo LC, Garcia MI, Taleb M, Euyoque JA, Clark T, Skidmore J, Ruiz M, Dharkar-Surber S, Schultz J, Philis-Tsimikas A. Dulce Digital: An mHealth SMS-Based Intervention Improves Glycemic Control in Hispanics With Type 2 Diabetes. Diabetes Care 2017;40(10):1349-1355. PMID:28600309

Goodarzi M, Ebrahimzadeh I, Rabi A, Saedipoor B, Jafarabadi MA. Impact of distance education via mobile phone text messaging on knowledge, attitude, practice and self efficacy of patients

with type 2 diabetes mellitus in Iran. Journal of Diabetes and Metabolic Disorders 2012;11(1):1-8. PMID:23497632
Goode AD, Winkler EAH, Reeves MM, Eakin EG. Relationship between intervention dose and outcomes in living well with diabetes—a randomized trial of a telephone-delivered lifestyle-based weight loss intervention. Am J Health Promot 2015;30(2):120-129. PMID:25372235
Gordon HS, Solanki P, Bokhour BG, Gopal RK. "I'm Not Feeling Like I'm Part of the Conversation" Patients' Perspectives on Communicating in Clinical Video Telehealth Visits. J Gen Intern Med;2020(Feb). PMID:32016705
Greenwood DA, Blozis SA, Young HM, Nesbitt TS, Quinn CC. Overcoming Clinical Inertia: A Randomized Clinical Trial of a Telehealth Remote Monitoring Intervention Using Paired Glucose Testing in Adults With Type 2 Diabetes. J Med Internet Res 2015;17(7):e178. PMID:26199142
Greenwood DA, Young HM, Quinn CC. Telehealth Remote Monitoring Systematic Review: Structured Self-monitoring of Blood Glucose and Impact on A1C. J Diabetes Sci Technol 2014;8(2):378-389. PMID:24876591
Hanley J, Fairbrother P, McCloughan L, Pagliari C, Paterson M, Pinnock H, Sheikh A, Wild S, McKinstry B. Qualitative study of telemonitoring of blood glucose and blood pressure in type 2 diabetes. BMJ Open 2015;5(12):e008896. PMID:26700275
Hansen CR, Perrild H, Koefoed BG, Zander M. Video consultations as add-on to standard care among patients with type 2 diabetes not responding to standard regimens: a randomized controlled trial. Eur J Endocrinol 2017;176(6):727-736. PMID:28325823
Hsu WC, Lau KHK, Huang R, Ghiloni S, Le H, Gilroy S, Abrahamson M, Moore J. Utilization of a Cloud-Based Diabetes Management Program for Insulin Initiation and Titration Enables Collaborative Decision Making Between Healthcare Providers and Patients. Diabetes Technol Ther 2016;18(2):59-67. PMID:26645932
Huang Z, Tao H, Meng Q, Jing L. Management of endocrine disease. Effects of telecare intervention on glycemic control in type 2 diabetes: a systematic review and meta-analysis of randomized controlled trials. Eur J Endocrinol 2015;172(3):R93-101. PMID:25227131
Jalil S, Myers T, Atkinson I. A meta-synthesis of behavioral outcomes from telemedicine clinical trials for type 2 diabetes and the Clinical User-Experience Evaluation (CUE). J Med Syst 2015;39(3):28. PMID:25677954
Jeong JY, Jeon JH, Bae KH, Choi YK, Park KG, Kim JG, Won KC, Cha BS, Ahn CW, Kim DW, Lee CH, Lee IK. Smart Care Based on Telemonitoring and Telemedicine for Type 2 Diabetes Care: Multi-Center Randomized Controlled Trial. Telemed J E Health 2018;24(8):604-613. PMID:29341843
Jha S, Dogra S, Yadav A, Siddiqui S, Panda M, Srivastava K, Raghuvanshi L, Kaur S, Bhargava A, Mathur R, Gupta SK, Waghdhare S. A prospective observational study to assess the effectiveness of an electronic health (E-health) and mobile health (M-health) platform versus conventional care for the management of diabetes mellitus. International Journal of Diabetes in Developing Countries 2016;36(4):529-534. doi:10.1007/s13410-016-0501-x
Jia H, Chuang H-C, Wu SS, Wang X, Chumbler NR. Long-term effect of home telehealth services on preventable hospitalization use. Journal of Rehabilitation Research and Development 2009;46(5):557-565. doi:10.1682/JRRD.2008.09.0133
Kempf K, Altpeter B, Berger J, Reuss O, Fuchs M, Schneider M, Gartner B, Niedermeier K, Martin S. Efficacy of the Telemedical Lifestyle intervention Program TeLiPro in Advanced Stages of Type 2 Diabetes: A Randomized Controlled Trial. Diabetes Care 2017;40(7):863-871. PMID:28500214
Kesavadev J, Shankar A, Pillai PBS, Krishnan G, Jothydev S. Cost-Effective Use of Telemedicine and Self-Monitoring of Blood Glucose via Diabetes Tele Management System (DTMS) to Achieve Target Glycosylated Hemoglobin Values Without Serious Symptomatic Hypoglycemia in

1,000 Subjects with Type 2 Diabetes Mellitus-A Retrospective Study. Diabetes Technol Ther 2012;14(9):772-776. PMID:22734662
Kim HS, Sun C, Yang SJ, Sun L, Li F, Choi IY, Cho J-H, Wang G, Yoon K-H. Randomized, Open-Label, Parallel Group Study to Evaluate the Effect of Internet-Based Glucose Management System on Subjects with Diabetes in China. Telemed J E Health 2016;22(8):666-674. PMID:26938489
Kim SI, Kim HS. Effectiveness of mobile and internet intervention in patients with obese type 2 diabetes. Int J Med Inform 2008;77(6):399-404. PMID:17881285
Kim Y, Park JE, Lee BW, Jung CH, Park DA. Comparative effectiveness of telemonitoring versus usual care for type 2 diabetes: A systematic review and meta-analysis. J Telemed Telecare 2018;25(10):587-601. PMID:30012042
Kongstad MB, Valentiner LS, Ried-Larsen M, Walker KC, Juhl CB, Langberg H. Effectiveness of remote feedback on physical activity in persons with type 2 diabetes: A systematic review and meta-analysis of randomized controlled trials 2019;25(1). PMID:28958212
Koopman RJ, Wakefield BJ, Johanning JL, Keplinger LE, Kruse RL, Bomar M, Bernt B, Wakefield DS, Mehr DR. Implementing home blood glucose and blood pressure telemonitoring in primary care practices for patients with diabetes: lessons learned. Telemed J E Health 2014;20(3):253-260. PMID:24350806
Lee JY, Chan CKY, Chua SS, Ng CJ, Paraidathathu T, Lee KKC, Lee SWH. Telemonitoring and Team-Based Management of Glycemic Control on People with Type 2 Diabetes: a Cluster-Randomized Controlled Trial. J Gen Intern Med 2020;35(1):87-94. PMID:31512187
Lee JY, Chan CKY, Chua SS, Paraidathathu T, Lee KK-C, Tan CSS, Nasir N, Lee SWH. Using telemedicine to support care for people with type 2 diabetes mellitus: A qualitative analysis of patients' perspectives. BMJ Open 2019;9(10). doi:10.1136/bmjopen-2018-026575
Lee PA, Greenfield G, Pappas Y. Patients' perception of using telehealth for type 2 diabetes management: a phenomenological study. BMC Health Serv Res 2018;18(1):549. PMID:30005696
Lee PA, Greenfield G, Pappas Y. The impact of telehealth remote patient monitoring on glycemic control in type 2 diabetes: a systematic review and meta-analysis of systematic reviews of randomised controlled trials. BMC Health Serv Res 2018;18(1):495. PMID:29940936
Lee SWH, Chan CKY, Chua SS, Chaiyakunapruk N. Comparative effectiveness of telemedicine strategies on type 2 diabetes management: A systematic review and network meta-analysis. Sci Rep 2017;7(1):12680. PMID:28978949
Lewinski AA, Patel UD, Diamantidis CJ, Oakes M, Baloch K, Crowley MJ, Wilson J, Pendergast J, Biola H, Boulware LE, Bosworth HB. Addressing Diabetes and Poorly Controlled Hypertension: Pragmatic mHealth Self-Management Intervention. J Med Internet Res 2019;21(4):e12541. PMID:30964439
Lim S, Kang SM, Kim KM, Moon JH, Choi SH, Hwang H, Jung HS, Park KS, Ryu JO, Jang HC. Multifactorial intervention in diabetes care using real-time monitoring and tailored feedback in type 2 diabetes. Acta Diabetol 2016;53(2):189-198. PMID:25936739
Liou JK, Soon MS, Chen CH, Huang TF, Chen YP, Yeh YP, Chang CJ, Kuo SJ, Hsieh MC. Shared care combined with telecare improves glycemic control of diabetic patients in a rural underserved community. Telemed J E Health 2014;20(2):175-178. PMID:24320193
Luley C, Blaik A, Reschke K, Klose S, Westphal S. Weight loss in obese patients with type 2 diabetes: effects of telemonitoring plus a diet combination - the Active Body Control (ABC) Program. Diabetes Res Clin Pract 2011;91(3):286-292. PMID:21168231
Maslakpak MH, Razmara S, Niazkhani Z. Effects of Face-to-Face and Telephone-Based Family-Oriented Education on Self-Care Behavior and Patient Outcomes in Type 2 Diabetes: A Randomized Controlled Trial. J Diabetes Res 2017;2017(8404328). doi:10.1155/2017/8404328

McFarland M, Davis K, Wallace J, Wan J, Cassidy R, Morgan T, Venugopal D. Use of home telehealth monitoring with active medication therapy management by clinical pharmacists in veterans with poorly controlled type 2 diabetes mellitus. Pharmacotherapy 2012;32(5):420-426. PMID:22488512

McMahon GT, Fonda SJ, Gomes HE, Alexis G, Conlin PR. A randomized comparison of online- and telephone-based care management with internet training alone in adult patients with poorly controlled type 2 diabetes. Diabetes Technol Ther 2012;14(11):1060-1067. PMID:22953754

Mushcab H, Kernohan WG, Wallace J, Martin S. Web-Based Remote Monitoring Systems for Self-Managing Type 2 Diabetes: A Systematic Review. Diabetes Technol Ther 2015;17(8):498-509. PMID:25830528

Nicolucci A, Cercone S, Chiriatti A, Muscas F, Gensini G. A Randomized Trial on Home Telemonitoring for the Management of Metabolic and Cardiovascular Risk in Patients with Type 2 Diabetes. Diabetes Technol Ther 2015;17(8):563-570. PMID:26154338

Odnoletkova I, Goderis G, Nobels F, Fieuws S, Aertgeerts B, Annemans L, Ramaekers D. Optimizing diabetes control in people with Type 2 diabetes through nurse-led telecoaching. Diabet Med 2016;33(6):777-785. PMID:26872105

Parsons SN, Luzio SD, Harvey JN, Bain SC, Cheung WY, Watkins A, Owens DR. Effect of structured self-monitoring of blood glucose, with and without additional TeleCare support, on overall glycaemic control in non-insulin treated Type 2 diabetes: the SMBG Study, a 12-month randomized controlled trial. Diabet Med 2019;36(5):578-590. PMID:30653704

Plotnikoff RC, Karunamuni N, Courneya KS, Sigal RJ, Johnson JA, Johnson ST. The Alberta Diabetes and Physical Activity Trial (ADAPT): a randomized trial evaluating theory-based interventions to increase physical activity in adults with type 2 diabetes. Ann Behav Med 2013;45(1):45-56. PMID:22922954

Pressman AR, Kinoshita L, Kirk S, Barbosa GM, Chou C, Minkoff J. A novel telemonitoring device for improving diabetes control: protocol and results from a randomized clinical trial. Telemed J E Health 2014;20(2):109-114. PMID:24404816

Ramadas A, Chan CKY, Oldenburg B, Hussein Z, Quek KF. Randomised-controlled trial of a web-based dietary intervention for patients with type 2 diabetes: changes in health cognitions and glycemic control. BMC Public Health 2018;18(1):716. PMID:29884161

Rasmussen OW, Lauszus FF, Loekke M. Telemedicine compared with standard care in type 2 diabetes mellitus: A randomized trial in an outpatient clinic. J Telemed Telecare 2016;22(6):363-368. PMID:26468213

Robinson MD, Branham AR, Locklear A, Robertson S, Gridley T. Measuring Satisfaction and Usability of FaceTime for Virtual Visits in Patients with Uncontrolled Diabetes. Telemed J E Health 2016;22(2):138-143. PMID:26295592

Rodriguez-Idigoras MI, Sepulveda-Munoz J, Sanchez-Garrido-Escudero R, Martinez-Gonzalez JL, Escolar-Castello JL, Paniagua-Gomez IM, Bernal-Lopez R, Fuentes-Simon MV, Garofano-Serrano D. Telemedicine influence on the follow-up of type 2 diabetes patients. Diabetes Technol Ther 2009;11(7):431-437. PMID:19580356

Sarayani A, Mashayekhi M, Nosrati M, Jahangard-Rafsanjani Z, Javadi M, Saadat N, Najafi S, Gholami K. Efficacy of a telephone-based intervention among patients with type-2 diabetes; a randomized controlled trial in pharmacy practice. International Journal of Clinical Pharmacy 2018;40(2):345-353. PMID:29435911

Schechter CB, Walker EA, Ortega FM, Chamany S, Silver LD. Costs and effects of a telephonic diabetes self-management support intervention using health educators. J Diabetes Complications 2016;30(2):300-305. PMID:26750743

Steventon A, Bardsley M, Doll H, Tuckey E, Newman SP. Effect of telehealth on glycaemic control: analysis of patients with type 2 diabetes in the Whole Systems Demonstrator cluster randomised trial. BMC Health Serv Res 2014;14:334. PMID:25100190

Stone RA, Rao RH, Sevick MA, Cheng C, Hough LJ, Macpherson DS, Franko CM, Anglin RA, Obrosky DS, DeRubertis FR. Active care management supported by home telemonitoring in veterans with type 2 diabetes: the DiaTel randomized controlled trial. Diabetes Care 2010;33(3):478-484. PMID:20009091

Stone RA, Sevick MA, Rao RH, Macpherson DS, Cheng C, Kim S, Hough LJ, DeRubertis FR. The Diabetes Telemonitoring Study Extension: an exploratory randomized comparison of alternative interventions to maintain glycemic control after withdrawal of diabetes home telemonitoring. J Am Med Inform Assoc 2012;19(6):973-979. PMID:22610495

Swoboda C.M., Miller C.K., Wills C.E. Impact of a goal setting and decision support telephone coaching intervention on diet, psychosocial, and decision outcomes among people with type 2 diabetes. Patient Education and Counseling 2017;100(7):1367-1373. PMID:28215827

Tang PC, Overhage JM, Chan AS, Brown NL, Aghighi B, Entwistle MP, Hui SL, Hyde SM, Klieman LH, Mitchell CJ, Perkins AJ, Qureshi LS, Waltimyer TA, Winters LJ, Young CY. Online disease management of diabetes: engaging and motivating patients online with enhanced resources-diabetes (EMPOWER-D), a randomized controlled trial. J Am Med Inform Assoc 2013;20(3):526-534. PMID:23171659

Tavsanly NG, Karadakova A, Saygili F. The use of videophone technology (telenursing) in the glycaemic control of diabetic patients: a randomized controlled trial. J Diab Res Clin Met 2013;2(1):1.

Tildesley HD, Mazanderani AB, Ross SA. Effect of Internet therapeutic intervention on A1C levels in patients with type 2 diabetes treated with insulin. Diabetes Care 2010;33(8):1738-1740. PMID:20668152

Tildesley HD, Wright AM, Chan JHM, Mazanderani AB, Ross SA, Tildesley HG, Lee AM, Tang TS, White AS. A comparison of internet monitoring with continuous glucose monitoring in insulin-requiring type 2 diabetes mellitus. Can J Diabetes 2013;37(5):305-308. PMID:24500556

Timmerberg BD, Wurst J, Patterson J, Spaulding RJ, Belz NE. Feasibility of using videoconferencing to provide diabetes education: a pilot study. J Telemed Telecare 2009;15(2):95-97. PMID:19246610

Toledo FGS, Ruppert K, Huber KA, Siminerio LM. Efficacy of the Telemedicine for Reach, Education, Access, and Treatment (TREAT) model for diabetes care. Diabetes Care 2014;37(8):e179-80. PMID:25061149

Trief PM, Fisher L, Sandberg J, Cibula DA, Dimmock J, Hessler DM, Forken P, Weinstock RS. Health and Psychosocial Outcomes of a Telephonic Couples Behavior Change Intervention in Patients With Poorly Controlled Type 2 Diabetes: A Randomized Clinical Trial. Diabetes Care 2016;39(12):2165-2173. PMID:27456837

Varney JE, Liew D, Weiland TJ, Inder WJ, Jelinek GA. The cost-effectiveness of hospital-based telephone coaching for people with type 2 diabetes: a 10 year modelling analysis. BMC Health Serv Res 2016;16(1):521. PMID:27678079

Varney JE, Weiland TJ, Inder WJ, Jelinek GA. Effect of hospital-based telephone coaching on glycaemic control and adherence to management guidelines in type 2 diabetes, a randomised controlled trial. Internal Medicine Journal 2014;44(9):890-897. PMID:24963611

Vasconcelos HCA de, Lira Neto JCG, Araujo MFM de, Carvalho GCN, Souza Teixeira CR de, Freitas R de, Damasceno MMC. Telecoaching programme for type 2 diabetes control: a randomised clinical trial. Br J Nurs 2018;27(19):1115-1120. PMID:30346819

Von Storch K, Graaf E, Wunderlich M, Rietz C, Polidori MC, Woopen C. Telemedicine-Assisted Self-Management Program for Type 2 Diabetes Patients. Diabetes Technol Ther 2019;21(9):514-521. PMID:31287736

Wakefield BJ, Holman JE, Ray A, Scherubel M, Adams MR, Hillis SL, Rosenthal GE. Effectiveness of home telehealth in comorbid diabetes and hypertension: a randomized, controlled trial. Telemed J E Health 2011;17(4):254-261. PMID:21476945

Wakefield BJ, Holman JE, Ray A, Scherubel M, Adams MR, Hills SL, Rosenthal GE. Outcomes of a home telehealth intervention for patients with diabetes and hypertension. Telemed J E Health 2012;18(8):575-579. PMID:22873700

Wakefield BJ, Koopman RJ, Keplinger LE, Bomar M, Bernt B, Johanning JL, Kruse RL, Davis JW, Wakefield DS, Mehr DR. Effect of home telemonitoring on glycemic and blood pressure control in primary care clinic patients with diabetes. Telemed J E Health 2014;20(3):199-205. PMID:24404819

Walker EA, Shmukler C, Ullman R, Blanco E, Scollan-Koliopoulus M, Cohen HW. Results of a successful telephonic intervention to improve diabetes control in urban adults: A randomized trial. Diabetes Care 2011;34(1):2-7. PMID:21193619

Wang G, Zhang Z, Feng Y, Sun L, Xiao X, Wang G, Gao Y, Wang H, Zhang H, Deng Y, Sun C. Telemedicine in the Management of Type 2 Diabetes Mellitus. Am J Med Sci 2017;353(1):1-5. PMID:28104096

Warren R, Carlisle K, Mihala G, Scuffham PA. Effects of telemonitoring on glycaemic control and healthcare costs in type 2 diabetes: A randomised controlled trial. J Telemed Telecare 2018;24(9):586-595. PMID:28814128

Wild SH, Hanley J, Lewis SC, McKnight JA, McCloughan LB, Padfield PL, Parker RA, Paterson M, Pinnock H, Sheikh A, McKinstry B. Supported Telemonitoring and Glycemic Control in People with Type 2 Diabetes: The Telescot Diabetes Pragmatic Multicenter Randomized Controlled Trial. PLoS Med 2016;13(7):e1002098. PMID:27458809

Wu L, Forbes A, While A. Patients' experience of a telephone booster intervention to support weight management in Type 2 diabetes and its acceptability. J Telemed Telecare 2010;16(4):221-223. PMID:20511580

Zhai YK, Zhu WJ, Cai YL, Sun DX, Zhao J. Clinical- and cost-effectiveness of telemedicine in type 2 diabetes mellitus: a systematic review and meta-analysis. Medicine (Baltimore) 2014;93(28):e312. PMID:25526482

Zhou P, Xu L, Liu X, Huang J, Xu W, Chen W. Web-based telemedicine for management of type 2 diabetes through glucose uploads: a randomized controlled trial. Int J Clin Exp Pathol 2014;7(12):8848-8854. PMID:25674254
