# Supplementary material for: Effect of Telemetric Interventions on Glycated Hemoglobin A1c and Management of Type 2 Diabetes Mellitus: Systematic Meta-Review
Source: J Med Internet Res. 2021 Feb 17;23(2):e23252. doi: 10.2196/23252 (PMC7929744; doi:10.2196/23252)
Supplement: Multimedia Appendix 4 [file jmir_v23i2e23252_app4.pdf]

Summary of the studies included in this systematic meta-review.

Study	Topic	Design	Participants/ included studies	Intervention/Control	Duration (Months)	Outcomes	Author's conclusions
**Systematic reviews and meta-analysis (n=10)**							
**(Mushcab et al. 2015)**<br><br>**South Korea, US, UK, Taiwan, Spain, Poland, India**	Web–based remote monitoring systems for self-managing type 2 diabetes	SR	n=19; (10 quasi-experimental & 9 RCTs)<br><br>Patients (n=3032)	Web–based remote monitoring systems	-	• 15 studies showed positive improvement in HbA1c levels<br>• One study showed high acceptance of the technology among participants	Not conclusive:<br><br>Optimal design of a telemedicine system is still uncertain, and the value of the real-time blood glucose transmissions is still controversial
**(Lee et al. 2018)**<br><br>**-**	Impact of telehealth remote patient monitoring on glycemic control	SR & MA of SRs of RCTs	SRs (n=4) reporting 29 studies<br><br>Patients (n=10531)	Data transmission: internet/web, automatic transmission, automatic mobile transmission, telephone<br><br>Feedback methods: automated message from computer, human calls, human message, videoconferencing (messages include internet, SMS, patient portal, telehealth system)	-	• Telehealth interventions produced a small but significant improvement in HbA1c levels compared with usual care (MD: -0.55, 95% CI: -0.73, − 0.36), P<0.00001<br>• Greatest effect was seen in telephone-delivered interventions, followed by internet blood glucose monitoring system interventions and lastly interventions involving automatic transmission of SMBG using a mobile phone or a telehealth unit	Mildly positive:<br><br>Telehealth is effective in controling HbA1c levels<br><br>Need for better quality primary studies as well as systematic reviews of RCTs
**(Greenwood et al. 2014)**<br><br>**US, Korea, Italy, Poland, Spain**	Telehealth remote monitoring systematic review: structured self-monitoring of blood glucose and impact on A1c	SR	RCTs (n=15)<br><br>Patients (n=3744)	RCTs incorporated 1 or more essential elements of SMBG identified by the International Diabetes Federation (patient education, provider education, structured SMBG profile, SMBG goals, feedback, data used to modify treatment, interactive communication or shared decision making)<br><br>Data transmission via telephone, computer, internet–based platform<br>Feedback methods: telephone calls, videoconferencing, SMS, text messaging, secure messages via patient portal or telehealth system	-	• Research incorporating 5 of the 7 elements consistently achieved significant A1c improvements between study groups<br>• Interventions using more SMBG elements are associated with an improvement in A1c<br>• Studies with the largest A1c decrease incorporated 6 of the 7 elements and computer decision support<br>• 2 studies with 5 of the 7 elements and active medication management achieved significant A1c decreases	Positive:<br><br>Incorporating more elements of structured SMBG is associated with improved A1c
**(Lee et al. 2017)**<br><br>**Mainly North America**	Comparative effectiveness of telemedicine strategies on diabetes management	SR & network MA	RCTs (n=107);<br><br>Patients (n=20501)	Teleeducation, telecase-management, telementoring, teleconsultation<br>Medium of communication: telephone (42%), the internet (34%), mobile phones (13%), SMS (9%), videoconferencing (7%), computer (4%) pagers (1%)	-	• Over a median of 6 months follow-up, telemedicine reduced HbA1c by a mean of 0.43% (95% CI: −0.64% to −0.21%), P<0.001<br>• All telemedicine strategies were effective in reducing HbA1c significantly compared to usual care except for telecase-management and telementoring, with mean difference ranging from 0.37% and 0.71%<br>• Ranking indicated that teleconsultation was the most effective telemedicine strategy, followed by telecase-management plus telemonitoring, and finally teleeducation plus telecase-management	Mildly Positive:<br><br>Most telemedicine strategies can be useful, either as an adjunct or to replace usual care, leading to clinically meaningful reduction in HbA1c

Study	Topic	Design	Participants/ included studies	Intervention/Control	Duration (Months)	Outcomes	Author's conclusions
**(Zhai et al. 2014)**<br><br>**-**	Clinical- and cost-effectiveness of telemedicine	SR & MA	RCTs (n=35)<br><br>Patients (n=8149)	12 studies: via telephone<br><br>19 studies: internet–based (website/videoconferencing)<br><br>4 studies: electronically transmitted recommendations made by clinician	-	• Small, but statistically significant, decrease in HbA1c following intervention, compared to CG (pooled difference in mean-s =-0.37, 95% CI=-0.49 to -0.25, Z=-6.08, P<0.001)<br>• Only 2 studies included assessment of cost-effectiveness; studies were disparate, both in terms of overall expense and relative cost-effectiveness	Mildly positive:<br><br>Optimization of telemedicine approaches could potentially allow for more effective self-management of disease in type 2 diabetes patients, though evidence to-date is unconvincing Significant publication bias was detected
**(Jalil et al. 2015)**<br><br>**-**	Behavioral outcomes from telemedicine clinical trials for type 2 diabetes and the clinical user-experience evaluation	Meta-Synthesis	Clinical Trials (n=19)<br><br>Patients (n=2979)	Technologies used: portable device, transfer of data via modem followed by telephone counseling, telephone–based system, voice message from clinician, interactive programs on CD Rom, web–based programs, teleconferencing	-	• Significantly results found that every clinical trial showed noteworthy positive behavioral outcomes of telemedicine<br>• Even though telemedicine does not improve medical conditions for certain, it does invoke positive behavioral change	Mildly positive:<br><br>Technology-intervened treatments provide positive behavior changes among patients and are potentially highly beneficial for diabetes management
**(Kim et al. 2018)**<br><br>**US, Korea, Italy, China, UK, Australia, Canada, Finland, Japan, Poland, Spain**	Comparative effectiveness of telemonitoring versus usual care	SR & MA	n=38<br><br>Patients (n=6855)	Telemonitoring interventions; transmission of biological information via information communication technologies	-	• Telemonitoring was associated with a significant decrease in HbA1c levels compared to usual care (WMD –0.42%, 95% CI –0.56 to –0.27), P<0.001<br>• Telemonitoring group was associated with significantly lower BMI (WMD –0.25 kg/m2, 95% CI –0.49 to –0.01, I^2=16.7%) compared to usual care<br>• Telemonitoring reduced HbA1c levels in studies that monitored patients' medication adherence, provided counselling, education and alarm message<br>• Telemonitoring was associated with a significant HbA1c reduction when biological data were transmitted through a web–based device weekly, when voice feedback was performed daily or immediately and when patients were provided with counselling	Positive
**(Kongstad et al. 2019)**<br><br>**-**	Effectiveness of remote feedback on physical activity	SR and MA of RCTs	n=27<br><br>Patients (n=4215)	Remote feedback by telephone calls, webpage (text–based), e-mail, SMS	-	• Overall effect size in favour of remote feedback interventions compared to standardized treatment, standard mean difference = 0.33 (95% CI: 0.17 to 0.49), I^2=81.7%), P=0.015<br>• Effect on physical activity was only influenced by study size, with a larger effect in small studies	Mildly Positive:<br><br>Adding remote feedback to standardized treatments aimed at increasing physical activity showed a small to moderate additional increase in physical activity levels

Study	Topic	Design	Participants/ included studies	Intervention/Control	Duration (Months)	Outcomes	Author's conclusions
**(Cassimatis/ Kavanagh 2012)**<br><br>**-**	Effects of type 2 diabetes behavioural telehealth interventions on glycaemic control and adherence	SR of RCTs	n=14<br><br>Patients (n=2300)	Behavioural telehealth interventions (majority: regular relephone calls; one study video messages via mobile phone; two studies automated telephone disease management calls); all included diabetes education	-	• 4 studies reported significant improvements in glycaemic control<br>• 5 of 8 studies on dietary adherence reported significant treatment effects, as did 5 of 8 on physical activity, 4 of 9 on blood glucose self-monitoring, and 3 of 8 on medication adherence<br>• Behavioural telehealth interventions can significantly improve both glycaemic and diabetes self-care outcomes	Positive:<br><br>Behavioural telehealth shows promise in improving diabetes self-care, glycaemic control
**(Huang et al. 2015)**<br><br>**US, Korea, Iran, Spain, Poland**	Effects of telecare intervention on glycemic control in type 2 diabetes	SR & MA of RCTs	RCTs (n=18); Patients<br><br>(n=3798)	Telecare as an intervention (self-monitored transmission of glucometer data and feedback by health professionals, or automatic medical devices): internet–based, automated and telephone	-	• Telecare significantly improved the management of diabetes<br>• Mean HbA1c values were reduced by -0.54 (95% CI, -0.75 to -0.34; P<0.05)<br>• Mean fasting plasma glucose levels by -9.00 mg/dl (95% CI, -17.36 to -0.64; P=0.03)<br>• Mean PPG levels reduced by -52.86 mg/dl (95% CI, -77.13 to -28.58; P<0.05)<br>• Significant reduction in HbA1c levels was associated with Asian populations, small sample size, and telecare, and with those patients with baseline HbA1c greater than 8.0%	Positive:<br><br>Significant improvement in glycemic control
**"Real-time video interventions" (n=12)**							
**(Dy et al. 2013)**<br><br>**US**	Use of telemedicine to improve glycemic management in a skilled nursing facility	RCT, pilot	IG (n=23)<br>CG (n=11)	IG: usual care + weekly/biweekly tele-consultations with endocrinologist via videoconferencing<br><br>CG: usual care; not adequately described	6	• 7 of 10 insulin-treated intervention subjects had basal doses reduced (18–69%) compared with 2 of 11 control subjects (reduced 10% and 25%)<br>• Decrease in percentage of intervention participants with episodes of hypoglycemia (<80 mg/dL) over the previous month from baseline (42%) to end of study (22%) versus a rise in the control group (from 36% to 45%)<br>• Less hyperglycemia (>400 mg/dL) (intervention, from 33% to 22%; control, from 22% to 55%)<br>• No end-of-study A1c values >8.0% in the intervention group versus 44% in controls<br><br>(No significances reported)	Positive:<br><br>Telemedicine diabetes consultations to skilled nursing facilities can improve glycemic management
**(Timmerberg et al. 2009)**<br><br>**US**	Feasibility of using videoconferencing to provide diabetes education	RCT, pilot	IG (n=13)<br>CG (n=13)	Both groups: individual counselling sessions (diabetes education) provided by a dietician through telemedicine (videoconferencing)<br><br>IG: +2 additional follow-up video-conferencing sessions, 4 and 8 weeks later	4	• After 16 weeks, HbA1c decreased from 7.24% to 7.15% in the intervention group and from 6.70% to 6.51% in the control group<br>• CG showed a significantly larger fall in HbA1c levels than IG (P=0.043)<br>• Cholesterol decreased in both groups (not significantly)	Positive:<br><br>Results suggest that providing nutritional therapy via videoconferencing may be useful in assisting patients to manage their conditions

Study	Topic	Design	Participants/ included studies	Intervention/Control	Duration (Months)	Outcomes	Author's conclusions
**(Hansen et al. 2017)** **Denmark**	Video consultations as add-on to standard care among patients with type 2 diabetes not responding to standard regimens	RCT	IG (n=83) CG (n=82)	IG: clinic–based care + telemedicine; monthly videoconferences via tablet computer preceded by uploads of measurements, with a nurse<br><br>CG: clinic –based care: doctor's visits every 3–6 months	8	• After 8 months: significant reduction of HbA1c compared to that in CG (0.69% vs 0.18%, P=0.022)<br>• At six-month follow-up, the intergroup difference in HbA1c reduction was no longer significant<br>• No changes in BP, BMI, waist/hip ratio, lipids, creatinine or fasting glucose in relation to the study<br>• Any significant changes in mental or physical health scores in SF-36 were not encountered	Positive:<br><br>Video consultations preceded by uploading relevant measurements can lead to clinically and statistically significant improvements in glycemic control among patients who have not responded to standard regimens
**(Davis et al. 2010)** **US**	Telehealth improves diabetes self-management in an underserved community	RCT	IG (n=85) CG (n=80)	IG: Diabetes TeleCare: 13-session curriculum delivered using telehealth strategies: interactive videoconferencing by the self-management education team (a nurse and a dietitian)<br><br>CG: usual care consisted of one 20-min diabetes education session	12	• Significant reduction in HbA1c in IG from baseline to 6 and 12 months (9.4 ± 0.3, 8.3 ± 0.3, and 8.2 ± 0.4) compared with CG (8.8 ± 0.3, 8.6 ± 0.3, and 8.6 ± 0.3), 6 months P=0.003 and 12 months P=0.004<br>• LDL cholesterol was reduced at 12 months IG compared with IG (P=0.02)<br>• BP, BMI and waist circumference show improvements in IG, but not significant at any timepoint compared with CG	Positive:<br><br>Improved metabolic control and reduced cardiovascular risk in an ethnically diverse and rural population
**(Rasmussen et al. 2016)** **Denmark**	Telemedicine compared with standard care in type 2 diabetes mellitus	RCT	IG (n=18) CG (n=22)	IG: individual video consultations (average 4.1 consultations)<br><br>CG: individual clinic visits (average 3.8)	6	• Significant differences between groups in HbA1c (-15 vs -11 mmol/mol, P=0.023), mean blood glucose (-18 vs -13 mmol/mol, P=0.015) and in cholesterol (-7 vs -6, P=0.044)<br>• No differences in LDL (-4 vs -6), DBP (-1 vs -7), and SBP (0 vs -1) (all P>0.05)<br>• Weight IG -0.6kg vs. CG -1.7 kg (P=0.023)	Mildly Positive:<br><br>Telemedicine was a safe and available option with favourable outcomes
**(Toledo et al. 2014)** **US**	Efficacy of the Telemedicine for Reach, Education, Access, and Treatment (TREAT) Model for Diabetes Care	RCT	IG (n=31) CG (n=63)	IG: Telemedicine (2 videoconferencing consultations with endocrinologist; patients assisted by local diabetes educator)<br><br>CG: usual care; not adequately described	6	• IG experienced a significant improvement in HbA1c from baseline; improvement was statistically greater than that observed in CG (P<0.05, CG 8.9±0.4 to 8.1±0.2, IG 8.6±0.3 to 6.6±0.2)<br>• 93% of TREAT subjects adhered to SMBG recommendations during intervention<br>• Mean incidence of hypoglycemia was 1.7 events/30 days/person	Positive:<br><br>Telemedicine offers the potential to overcome geographical barriers to care in rural communities
**(Egede et al. 2018)** **US**	Effect of psychotherapy for depression via home telehealth on glycemic control in adults with type 2 diabetes	RCT	IG (n=42) CG (n=47)	Both: 8 weekly sessions of behavioural activation treatment (BAT); BAT = cognitive behaviour therapy for depression<br><br>IG: therapy via videophone<br><br>CG: sessions in person	12	• Baseline mean HbA1c for IG remained at 6.9 (55 mmol/mol) at 12 months, whereas baseline mean HbA1c for CG increased to 7.7 (61 mmol/mol)<br>• Longitudinal trajectories of model-derived mean HbA1c indicated a significant main effect of treatment group on mean A1c value at study end, difference between groups = -0.82, P=0.0061	Positive:<br><br>Telemedicine was superior in achieving lower mean A1c values

Study	Topic	Design	Participants/ included studies	Intervention/Control	Duration (Months)	Outcomes	Author's conclusions
**(Tavşanli et al. 2013)**<br><br>**Turkey**	The use of videophone technology (telenursing) in the glycaemic control of diabetic patients	RCT	IG (n=24)<br>CG (n=24)	IG: weekly video-conferences, diabetes management education; 2 face-to-face meetings<br><br>CG: 2 face-to-face visits; no education	6	• At 6-months, HbA1c levels of the individuals in IG were significinatly lowered by 0.49% in total, and the A1c levels of the individuals in the control group were higher by 0.17% in total (P=0.013)<br>• At 6 months, FBGs were lower in IG (159.48±40.71 mg/dL) compared to CG (173.03±65.07 mg/dL), but no significant difference (P>0.05)	Positive:<br><br>Videophone technology can be useful in the glycaemic control
**(Carlisle/ Warren 2013)**<br><br>**Australia**	Qualitative case study of telehealth for in-home monitoring	Qualitative	Health practitioners (n=8)<br><br>Patients (n=4)<br><br>Semi-structured interviews	Usual care + diabetes educator nurse via an in-home broadband communication device; 14 video-conferences	12	• Conditions for effective implementation: e.g. positive working relationships, clinical buy-in, motivation and commitment from implementation team and health practitioners, integration telehealth into daily practice, user friendly technology<br>• Patients reported positive health and social outcomes (achieved their goals)	Mildly positive:<br><br>Improved diabetes control, but more support is required to ensure sustainability and widespread implementation
**(Gordon et al. 2020)**<br><br>**US**	Perspectives on Communicating in Clinical Video Telehealth Visits	Qualitative	Patients (n=27)<br><br>Semi-structured interviews	Clinical Video Telehealth Visits	Not reported	• Advantages: better access to appointments, shorter travel time, less time in waiting room<br>• Concerns: perceptions that providers paid less attention to them, barriers to speaking up and asking questions, difficulty establishing a provider-patient relationship, difficulty finding opportunities to speak, concerned about accuracy of physical exam	Limited positiv:<br><br>Potential, but communication challenges
**(Robinson et al. 2016)**<br><br>**US**	Measuring satisfaction and usability of facetime for virtual visits	Qualitative	Patients (n=34)<br><br>Survey	iPad2 with video-conferencing capabilities using FaceTime<br><br>Data transmission daily using home telemonitor	3	• 65% of patients reported satisfaction using FaceTime for visits<br>• 76% of patients agreed that FaceTime was effective in improving diabetes<br>• Overall satisfaction with technology declined over the study period among members of the diabetes care team	Positive:<br><br>Overall positive ratings in terms of usability and satisfaction
**(Ciemins et al. 2011)**<br><br>**US**	Using telehealth to provide diabetes care to patients in rural montana	Non-randomized controlled trial	IG (n=118)<br>CG (n=88)	IG: telehealth technology consisted of videoconferencing; monthly visits<br><br>CG: monthly face-to-face visits<br><br>Both: diabetes education via "Promoting Realistic Individual Self-Management Diabetes Program"	36	• Patient diabetes care satisfaction rates increased 191% and 131% among IG and CG respectively (P=0.51)<br>• Increased self-reported blood glucose monitoring as instructed (97% Telehealth vs. 89%; P=0.63) and increased dietary adherence (244% Telehealth vs. 159%; P=0.86)<br>• Control of each vascular risk factor (HbA1c <7%, LDL-C < 100, or BP < 130/80) increased in both groups over time as did the proportion of patients with two or more controlled risk factors (not significant at end of study, P>0.05)<br>• No significant differences in diabetes knowledge, communication, self-efficacy and diabetes symptoms (P>0.05)	Positive:<br><br>Viable strategy for addressing the unique challenges faced by patients living in rural communities
**"Real-time audio interventions" (n=17)**							

Study	Topic	Design	Participants/ included studies	Intervention/Control	Duration (Months)	Outcomes	Author's conclusions
**(Benson et al. 2019)**<br><br>**US**	Impact of ENHANCED (diEtitiaNs Helping pAtieNts CarE for Diabetes) Telemedicine on care outcomes	RCT	IG (n=60)<br>CG (n=58)	IG: usual care + monthly telephone coaching (medical nutrition therapy; self-management education)<br><br>CG: usual care with primary care provider; may have included referral to diabetes education program	12	• Modest but significantly greater improvement in number of diabetes optimal care measures met at 1-year-follow-up was found in IG (3.7 vs 3.2 in the control group P=0.017)<br>○ Measures: BP <140/90 mmHg, Statin use, HbA1c < 8%, not using tobacco and taking daily aspirin<br>• Number of patients with BP < 140/90 and HbA1c <8% increased in IG, but no significant difference compared with CG (P=0.538 and P=0.702)<br>• IG had significantly greater medication use, with 2.5 and 2.2 higher odds (compared with CG) of taking a statin [95% CI, 1.0 to 6.24]) and aspirin [95% CI, 0.90 to 5.19] as appropriate, respectively	Positive
**(Vasconcelos et al. 2018)**<br><br>**Brazil**	Telecoaching programme for type 2 diabetes control	RCT	IG (n=16)<br>CG (n=15)	IG: coaching via telephone calls by researcher nurse; 12 biweekly telephone contacts<br><br>CG: usual care; not adequately described	6	• IG improved: abdominal circumference (98.56 to 95.75; P=0.001), systolic (130.25 to 125.87; P=0.171) and diastolic BP (72.12 to 71.12; P=0.640) and BMI (29.99 to 29.96; P=0.764); variables related to glycaemic control (fasting venous glucose (7.925 to 7.162; P=0.378) and HbA1c (8.0 to 7.21; P=0.052); and HDL levels (1.165 to 1.191; P=0.59)<br>• Changes in BMI in CG (P=0.014), AC in the IG (p=0.001), SBP in CG (P=0.042), SBP in CG vs. IG (P=0.03), and HbA1c in IG/CG (P=0.048/P=0.052) were significant<br>• None of the other intergroup differences were significant (P>0.05)	Positive:<br><br>Telecoaching as effective tool for diabetes management
**(Barton et al. 2018)**<br><br>**US**	Clinical inertia in a randomized trial of telemedicine–based chronic disease management	RCT	IG (n=182)<br>CG (not specified)	IG: nurse-administered telemedicine intervention (by telephone) - monthly self-management education from nurses via phone encounters (disease management including self-monitoring, medication; behavior change e.g. diet)<br><br>CG: usual care; not adequately described	12	• Intensification was not recommended in 67.5% of encounters in which hemoglobin A1c was above goal, 72.5% in which systolic BP was above goal, and 73.9% in which low-density lipoprotein cholesterol was above goa<br>• In each disease state, treatment intensification was more likely with poorer control	None:<br><br>Despite enhancing treatment adherence, intervention was unsuccessful in countering clinical inertia, likely explaining its lack of effect on CVD risk factors
**(Odnoletkova et al. 2016)**<br><br>**Belgium**	Optimizing diabetes control in people with type 2 diabetes through nurse-led telecoaching	RCT	IG (n=287)<br>CG (n=287)	IG: telecoaching; 5 telephone sessions<br><br>CG: usual care + DVD with educational material	6	• At 6 months, the between-group differences in favour of telecoaching were: HbA1c -2 (95% CI -4; -1) mmol/mol [-0.2 (95% CI -0.3;-0.1)%; P=0.003] overall; BMI -0.4 kg/m2 (95% CI -0.6; -0.1; P=0.003); total cholesterol -6 mg/dl (95% CI -11; -1, P=0.012); weight change: -1.1 kg (95% CI -1.9 to -0.4) (P=0.004)<br>• At 18 months, the difference in HbA1c was: -2 (95% CI -3;-0) mmol/mol [-0.2 (95% CI -0.3; -0.0)%; P=0.046] overall	Positive:<br><br>Nurse-led telecoaching improved glycaemic control, total cholesterol levels and BMI in people<br>12 months after intervention completion, sustained improvements in glycaemic control

Study	Topic	Design	Participants/ included studies	Intervention/Control	Duration (Months)	Outcomes	Author’s conclusions
**(Blackberry et al. 2013)**<br><br>**Australia**	Effectiveness of nurse led telephone coaching on glycaemic control of type 2 diabetes	RCT	IG (n=235)<br>CG (n=236)	IG: nurse led telephone coaching: 8 telephone and one face-to-face coaching episodes<br><br>CG: usual care; not adequately described	18	• At 18 months, glycaemic control did not differ significantly (mean difference 0.02, 95% CI −0.20 to 0.24, P=0.84) between groups<br>• Other biochemical and clinical outcomes were similar in both groups	None:<br><br>Ineffective
**(Varney et al. 2016)**<br><br>**Australia**	Cost-effectiveness of hospital–based telephone coaching	RCT	IG (n=47)<br>CG (n=47)	Telephone coaching - extrapolate outcomes collected at 6 months over a 10 year time horizon *(intervention description see Varney et al. 2014 below)*	6	• Intervention dominated the control condition in the base-case analysis, contributing to cost savings of $3327 per participant, along with non-significant improvements in quality-adjusted life expectancy (0.2 QALE) and life expectancy (0.3 years)	Positive:<br><br>Cost of delivering the telephone coaching intervention continuously, for 10 years, was fully recovered through cost savings and a trend towards net health benefits
**(Varney et al. 2014)**<br><br>**Australia**	Effect of hospital–based telephone coaching on glycaemic control and adherence to management guidelines	RCT	IG (n=47)<br>CG (n=47)	IG: usual care + telephone coaching by dietitian (for achieving treatment targets); 6 sessions<br><br>CG: usual care only; attended diabetes clinic 3–6 monthly	6	• Significant interaction effects observed between group and time at 6 months, demonstrating improvement in HbA1c, fasting glucose, diastolic blood pressure<br>• IG improved compliance and physical activity<br>• Intervention’s effect on these parameters was not sustained at 12 months	Mildly positive:<br><br>Improves glycaemic control but effects not maintained
**(Fernandes et al. 2016)**<br><br>**Brazil**	Evaluation of the telephone intervention in the promotion of diabetes self-care	RCT	IG (n=104)<br>CG (n=106)	IG: 6 telephone calls<br><br>CG: usual care; not adequately described	6	• Mean effect of self-care scores in IG was 1.03 to 1.78 higher than CG, with progressive and significant improvement (P<0.001)	Positive:<br><br>Beneficial effect on diabetes self-care
**(Trief et al. 2016)**<br><br>**US**	Health and psychosocial outcomes of a telephonic couples behavior change intervention	RCT	Couple Calls (CC) (n=104)<br><br>Individual Calls (IC) (n=94)<br><br>Diabetes Education (DE) (n=82)	All arms: self-management education (2 calls)<br><br>CC and IC: 10 additional behavior chance calls<br><br>CC: Partners actively involved in calls	12	• Significant A1c reductions for all (12 months: CC -0.47%, IC -0.52%, DE -0.57%), with no differences between arms<br>• Lowest tertile (7.5–8.2%), no change from baseline; middle tertile (8.3–9.2%), only CC led to significantly lower A1c level; and highest tertile (≥9.3%), significant improvement for all interventions<br>• For BMI, CC showed significant improvement at 12 months (-0.474, P=0.021) and was significantly lower than other groups<br>• CC and DE led to signficantly decreased waist circumference<br>• IC greater SBP improvement than DE at 8 months (P = 0.021), but no significant differences from baseline were observed for any intervention at any follow-up<br>• Compared with baseline, IC group mean DBP was significantly lower at 8 and 12 months; for the CC arm, only the 4-month DBP was significantly lower, and the DE group showed no differences	Positive:<br><br>For those with exceedingly high A1c levels, education alone was beneficial, but additional intervention is needed to achieve glycemic targets

Study	Topic	Design	Participants/ included studies	Intervention/Control	Duration (Months)	Outcomes	Author's conclusions
**(Goode et al. 2015)**<br><br>**Australia**	Relationship between intervention dose and outcomes in living well with diabetes - telephone-delivered weight loss intervention	RCT	IG (n=151)<br>CG (n=151)	IG: up to 27 telephone counseling calls (weekly for the first 4 weeks, every 2 weeks for 5 months, monthly for remaining 12 months)<br><br>CG: brief summary of their assessment via e-mail; standard education brochures on diabetes self-management	18	• Only previous diagnosis of depression/ anxiety had a statistically significant (P=0.008) association with call completion<br>• Call completion was significantly associated with weight loss (P<0.001) but not the other outcomes (P>0.05)<br>• Relative to low call completion, mean weight loss was greater in the high–call completion group by -3.3% (95% CI, -5.0% to -1.5%)	Positive:<br><br>Increased dose of intervention was associated with greater weight loss
**(Maslakpak et al. 2017)**<br><br>**Iran**	Effects of face-to-face and telephone–based family-oriented education on self-care behavior and patient outcomes	RCT	Group 1 (n=30)<br>Group 2 (n=30)<br>Group 3 (n=30)	Group 1: face-to-face education; classes twice a week in first month, once a week in second<br><br>Group 2: telephone–based education: twice a week in first and second months and once a week in the third<br><br>Group 3: control: paper–based educational materials	3	• Overall self-care scores in the intervention groups were significantly higher than that in CG (P=0.0001)<br>• Lipid profiles significantly improved in the interventions compared to CG (P<0.05)<br>• Comparing the 2 interventions showed better results for the face-to-face group regarding dietary adherence + physical activity, but the latter group had comparable results in blood glucose monitoring, foot care + cholesterol<br>• FBG and HbA1c decreasing trend in the intervention groups, but this change did not reach statistical significance	Mildly positive:<br><br>Potential value of low-cost telephone technology in delivering effective diabetes care
**(Sarayani et al. 2018)**<br><br>**Iran**	Efficacy of a telephone-–based intervention	RCT	IG (n=50)<br>CG (n=50)	IG: 16 telephone calls by pharmacist<br><br>CG: usual care; not adequately described<br><br>Both: Before random allocation, patients attended a live education session by pharmacists	3	• HbA1c was significantly improved in both groups at month-3 (6.97 ± 1.41 vs. 7.09 ± 1.78) + remained steady at month-9 (6.96 ± 1.44 vs. 7.26 ± 1.85)<br>• No significant difference in HbA1c level over the study period between study group (P=0.78)<br>• Lipid profile showed small improvements in IG, but not significant<br>• Adherence and self-care score improvement was significantly higher in IG at month-3 and were maintained at month-9 (P<0.05)	Mildly positive:<br><br>Medication adherence and self-care significantly improved Clinical outcomes might have been diluted due to the live diabetes education session
**(Schechter et al. 2016)**<br><br>**US**	Costs and effects of a telephonic diabetes self-management support intervention using health educators	RCT	IG (n=443)<br>CG (n=498)	IG: telephone/print; 4 or 8 telephone calls<br>CG: print only<br><br>Both: printed diabetes self-management support materials	12	• Intervention provided to 443 people at a net cost of $187.61 each<br>• Each percentage point of net A1c reduction achieved at a cost of $464.41<br>• Labor costs were largest component of costs, cost-effectiveness was most sensitive to wages paid to the health educators	Positive:<br><br>Moderate cost relative to gains achieved

Study	Topic	Design	Participants/ included studies	Intervention/Control	Duration (Months)	Outcomes	Author's conclusions
**(Swoboda et al. 2017)**<br><br>**US**	Impact of a goal setting and decision support telephone coaching intervention on diet, psychosocial, and decision outcomes	RCT	IG (n=37)<br>CG (n=17)	IG: 1 in-person support session followed by 7 biweekly telephone coaching calls<br><br>CG: information about local health care resources on same contact schedule	4	• Significant difference between groups for diabetes empowerment (P=0.045)<br>• Significant increase in diet quality, diabetes self-efficacy, and diabetes empowerment<br>• Significant decrease in diabetes distress and depressive symptoms (all P<0.05) in IG<br>• No significant differences between groups in the change in the Healthy Eating Index 2010, depressive symptoms, diabetes distress, or diabetes self-efficacy scores	Positive:<br><br>Telephone coaching can improve psychosocial outcomes related to diabetes self-management
**(Walker et al. 2011)**<br><br>**US**	Results of a successful telephonic intervention to improve diabetes control in urban adults	RCT	Telephone group (n=262)<br><br>Print group (n=264)	Telephone group: up to 10 calls at 4- to 6-week intervals from health educator<br><br>Print group: no calls; not adequately described	12	• Telephone group had mean ± SE decline in HbA1c of 0.23 ± 0.11% over 1 year compared with a rise of 0.13 ± 0.13% for the print group (P=0.04)<br>• After adjusting for baseline HbA1c, sex, age, and insulin use, the difference in HbA1c was 0.40% (95% CI 0.10–0.70, P=0.009)<br>• Improvement in medication adherence was associated (P=0.005) with the telephonic intervention, but only among those not taking insulin<br>• Having at least 6 completed phone calls was associated with significant improvement in HbA1c	Mildly positive:<br><br>Successful in significantly, albeit modestly, improving diabetes control compared with a print intervention
**(Wu et al. 2010)**<br><br>**UK**	Patients' experience of a telephone booster intervention to support weight management	Qualitative	Patients (n=25)<br><br>Semi-structured Exit-interviews	Weekly telephone calls which repeated the key messages given in the clinic	3	• Patients were satisfied or very satisfied with telephone calls and most would recommend the intervention to others<br>• Benefits included: being reminded to comply with their regimen; prompting and motivating adherence to diabetes self-care behaviours; improved self-esteem; and feeling 'worthy of interest'	Positive:<br><br>The convenience and low cost of telephone support has much potential
**"Real-time audio + asynchronous intervention" (n=1)**							

Study	Topic	Design	Participants/ included studies	Intervention/Control	Duration (Months)	Outcomes	Author's conclusions
**(McMahon et al. 2012)**<br><br>**US**	A randomized comparison of online- and telephone- –based care management with internet training alone in adult patients with poorly controlled diabetes	RCT	Online care management (n=51)<br><br>Telephone–based care management (n=51)<br><br>Web training (n=49)	Online group: biweekly login to patient portal (internet–based); feedback via internal messaging system<br><br>Telephone group: biweekly calls<br><br>Web training group: usual care + online diabetes self-management resources but no care management support	12	• A1c declined significantly in all groups over 12 months<br>• A1c declined linearly at a rate of 0.32% (P<0.0001) per quarter for online group, 0.36% (P<0.0001) for telephone group, and 0.41% for web training group (P<0.0001)<br>• Rate of change over time did not differ significantly between groups (P=0.12 for the online care group compared with the Web training group and P=0.35 for the telephone care group compared with the Web training group), suggesting no significant intervention<br>• Blood pressure: significant decrease in telephone group, P=0.006 systolic and P=0.001 diastolic<br>• Weight: non-significant increase in all groups<br>• BMI: non-significant increase in all groups	Neutral:<br><br>Online, telephone–based and web training were each associated with a substantial improvement in A1c internet access and training alone may be as effective as care management
**"Asynchronous interventions" (n=28)**							
**(Cho et al. 2017)**<br><br>**South Korea**	internet–based health gateway device for interactive communication and automatic data uploading	RCT	IG (n=244)<br>CG (n=240)	IG: internet–based communication via gateway device (feedback + data transmission); for the first 3 months, nurses sent a recommendation every week, and then, for the last 3 months, every other week<br><br>CG: usual care; not adequately described	6	• Upon six-month follow-up, HbA1c levels were significantly decreased from 7.86±0.69% to 7.55±0.86% within the IG (P<0.001) compared to 7.81±0.66% to 7.70±0.88% within the CG, significant difference between groups (P=0.0102)<br>• A subgroup with baseline HbA1c higher than 8% and good compliance achieved a reduction of HbA1c by 0.8±1.05%<br>• Glucose control and waist circumference reduction were more effective in females and subjects older than 40 years of age<br>• No adverse events	Positive:<br><br>E-health care system was effective for glucose control and body composition improvement
**(Ramadas et al. 2018)**<br><br>**Malaysia**	Web–based dietary intervention for patients with type 2 diabetes: changes in health cognitions and glycemic control	RCT	IG (n=62)<br>CG (n=66)	IG: Web–based dietary intervention (Telemedicine) in addition to usual care: recommendations delivered via the study website; 12 lesson plan<br><br>CG: usual care; not adequately described	6	• Margin of improvement in mean Dietary Knowledge, Attitude and Behaviour score in IG was larger than CG at postintervention (11.1 ± 0.9 vs. 6.5 ± 9.4, P<0.001) and follow-up (19.8 ± 1.1 vs. 7.6 ± 0.7,P<0.001), as compared to the baseline<br>• No significant difference between intervention and control arms with respect to Dietary Stages of Change score and glycaemic control<br>• IG showed improved this score (199.7 ± 18.2 vs193.3 ± 14.6, P=0.046), FBG (7.9 ± 2.5 mmol/L vs. 8.9 ± 3.9 mmol/L, P=0.015) and HbA1c (8.5 ± 1.8% vs. 9.1 ± 2.0%, P=0.004) at follow-up compared to the baseline, whereas such improvement was not seen in CG (no significant differences between groups in HbA1c and FBG)	Mildly positive:<br><br>Most important impact was on the overall Dietary Knowledge, Attitude and Behaviour score

Study	Topic	Design	Participants/ included studies	Intervention/Control	Duration (Months)	Outcomes	Author's conclusions
**(Egede et al. 2017)**<br><br>**US**	Technology-assisted case management in low income adults	RCT	IG (n=54)<br>CG (n=59)	IG: technology-assisted case management, web–based; data transmission daily via website; nurse case manager made medication adjustments weekly (for patients on insulin) or biweekly (for patients on oral agents) under supervision of physicians<br><br>CG: usual care; not adequately described	6	• HbA1c at 6 months for technology-assisted case management was significantly lower compared to the usual care group (-0.99, P=0.024).<br>• Rate of decline in HbA1c over time for IG was significantly faster compared to the usual care group (-0.16, P=0.038)	Positive:<br><br>Technology-assisted case management by a nurse with medication titration under physician supervision is efficacious in improving glycemic control
**(Tildesley et al. 2013)**<br><br>**Canada**	A comparison of internet monitoring with continuous glucose monitoring in insulin-requiring type 2 diabetes mellitus	RCT	RT-CGM (n=25)<br>IBGMS (n=25)	Real-time continuous glucose monitoring (RT-CGM):<br>Every 2 weeks data transmission via e-mail; feedback by endocrinologist<br><br>Internet blood glucose monitoring system (IBGMS):<br>Every 2 weeks web–based data tansmission<br>Endocrinologist reviewed and sent feedback through web–based system<br><br>Both: 2 endocrinologist visits	6	• After 6-month follow-up, both groups showed significant within-group improvements in A1c level<br>• In IBGMS group, A1c level decreased from 8.79%±1.25% to 7.96%±1.30% (P<0.05)<br>• RT-CGM group decreased from 8.80%±1.37% to 7.49%±0.70% (P<0.001)<br>• IBGMS and RT-CGM did not show significantly different A1c levels at baseline, 3 and 6 months (P>0.05)	Mildly positive:<br><br>Use of both IBGMS and RT-CGM significantly improved A1c levels; No significant differences in A1c values between groups after 6 months
**(Cho et al. 2011)**<br><br>**Korea**	Effects on diabetes management of a health care Provider mediated, remote coaching system via a PDA-type glucometer and the internet	RCT	IG (n=35)<br>CG (n=36)	IG: nurse sent measured data/patient information; physician: feedback once a week via internet; replied to questions within 24 hours<br><br>CG: general diabetes education by nurse; no recommendations by physician	3	• Compared with baseline, HbA1c was significantly reduced at three-month follow-up in IG (8.0% vs. 7.5%; P<0.01), but not in CG<br>• Total cholesterol was significantly reduced in IG (10.7 mmol/L vs. 10.4 mmol/L; P=0.043)	Positive:<br><br>New system could be implemented widely and would contribute to improving the quality of diabetes care
**(Avdal et al. 2011)**<br><br>**Turkey**	Effects of web–based diabetes education on diabetes care results	RCT	IG (n=61)<br>CG (n=61)	IG: web–based education; data transmission and feedback via web<br><br>CG: education in polyclinic by nurse	6	• A1c levels of the individuals with diabetes who were monitored through the Web decreased (t =6.63; P<0.05), and the rate of attending health check visits increased (z=5.97; P<0.05), while no difference was detected in the control group (t = -0.63; P=0.534; z = -0.80; P=426)	Positive:<br><br>Web use could be adopted as a complementary tool for monitoring individuals with diabetes
**(Tildesley et al. 2010)**<br><br>**Canada**	Effect of internet therapeutic intervention on A1c levels in patients with type 2 diabetes treated with insulin	RCT	IG (n=25)<br>CG (n=25)	IG: internet–based glucose monitoring system; Intervention group uploaded blood glucose readings every 2 weeks to a secure Website for feedback from endocrinologist<br><br>CG: usual care; diary of SMBG	6	• Over a 6-month follow up, A1c dropped from 8.8 to 7.6% (P<0.001) in the IG compared with 8.5 to 8.4% (P=0.51) in the CG<br>• Difference between the two groups at 6 months postintervention was statistically significant even after adjusting for baseline A1c levels (P<0.05)	Positive:<br><br>Use of IBGMS significantly improved A1c levels

Study	Topic	Design	Participants/ included studies	Intervention/Control	Duration (Months)	Outcomes	Author's conclusions
**(Lee et al. 2020)**<br><br>**Malaysia**	Telemonitoring and team–based management of glycemic control on people with type 2 diabetes	RCT	IG (n=120)<br>CG (n=120)	IG: automatically data transmission to online portal (6 glucose readings/ week); automated feedback on glycemic and metabolic results; monthly communications from research team (education, motivation); 3 clinic visits<br><br>CG: usual care; not adequately described	ca. 12	• IG reported larger improvements in glycemic control compared with CG at the end of study (week 24, − 0.05%; 95% CI − 0.10 to 0.00%, P>0.05) and at follow-up (week 52, − 0.03%; − 0.07 to 0.02%, P=0.226)<br>• Evidence was lacking for any between-group differences in other outcomes including Problem Area in Diabetes questionnaire, Diabetes Knowledge Test, and cholesterol levels	Limited benefit of replacing telemedicine with the current practice of SMBG
**(Wild et al. 2016)**<br><br>**UK**	Supported telemonitoring and glycemic control in people with type 2 diabetes	RCT	IG (n=160)<br>CG (n=161)	IG: Telemonitoring (self-measurement and transmission to website of glucose for weekly review by clinicians)<br><br>CG: Individual usual care	9	• Mean HbA1c at follow-up was 63.0 (15.5) mmol/mol in the IG and 67.8 (14.7) mmol/mol in the CG<br>• Adjusted mean HbA1c was 5.60mmol/mol / 0.51% lower (95%CI 2.38 to 8.81mmol/mol/ 95%CI 0.22%to 0.81%, P=0.0007)<br>• Adjusted mean ambulatory systolic BP was 3.06mmHg lower (95%CI 0.56– 5.56mmHg, p = 0.017) and mean ambulatory diastolic BP was 2.17mmHg lower (95%CI 0.62–3.72, P=0.006) among people in the IG when compared with CG<br>• Greater number of telephone calls between practice nurses and patients in IG compared with CG (rate ratio 7.50 (95%CI 4.45–12.65, P<0.0001) but no other significant differences between groups in use of health services	Positive:<br><br>Supported telemonitoring resulted in clinically important improvements in control of glycaemia in patients with type 2 diabetes in family practice
**(Cho et al. 2011)**<br><br>**Korea**	Glucose data-filtering system with automatic response software to reduce the physician workload	RCT	IG (n=36)<br>CG (n=39)	IG: software automatically filtered self-monitored blood glucose data; daily upload; communication only via internet with individualized electronic chart system<br><br>CG: Physicians had to contact patients manuelly and sent recommendations through the internet once every other week	6	• Physicians' log-in time was reduced by 67% and 55% in patients with HbA1c ≤6.5% and >6.5%, respectively (P<0.05)<br>• Patients with <6.5%: HbA1c levels were maintained at <6.5% during the study period in both groups (6.0 to 6.4% for the IG; 6.1 to 6.4% for the CG; P=0.01 for both)<br>• Patients with >6.5%: (7.3 to 7.7% in the IG, P=0.062; 7.4 to 7.7% in the CG, P=0.074)	Positive:<br><br>Efficacy and safety of the software for online communication could improve cost-effectiveness
**(Dario et al. 2017)**<br><br>**Italy**	Telemonitoring of type 2 diabetes Mellitus in Italy	RCT	IG (n=208)<br>CG (n=91)	IG: gateway for data transmission to a "Regional eHealth Center"; "Home Care Portal"<br><br>CG: paper log books, planned clinic visits	12	• No clinically important improvement in health-related QoL (some items significant improvement, but not sufficient to maintain that Telemedicine improves HRQoL)<br>• No statistically significant difference in HbA1c between the two groups (P=0.76; -0.2 in IG and -0.25 in CG)<br>• Outpatient visits and planned hospitalizations were significantly reduced in IG (P<0.0001 and P=0.02)	None

Study	Topic	Design	Participants/ included studies	Intervention/Control	Duration (Months)	Outcomes	Author's conclusions
**(Wakefield et al. 2011)**<br><br>**US**	Effectiveness of home telehealth in comorbid diabetes and hypertension	RCT	High-Intensity Intervention (HI) (n=93)<br><br>Low-Intensity intervention (LI) (n=102)<br><br>CG (n=107)	Home telehealth device (data transmission and automated responses) and nurse care management (nurse reviewed responses)<br><br>High-Intensity: study team developed branching disease management algorithm (algorithm focused on diet, exercise, smoking, foot care etc.)<br><br>Low-Intensity: No algorithm used<br>CG: usual care; not adequately described	6	• 6 Months: In HI (-0.44, P=0.0001) and LI (-0.40, P=0.0003) groups HbA1c decreased significantly<br>• 6 Months: HI and LI groups decreasing significantly more compared to CG (P=0.03 and 0.02, respectively)<br>• 12 months: no longer significant decrease in LI and HI (P=0.22 and 0.19)<br>• 6 months: significant decrease in SBP in HI (-6.05, P=0.01), significant difference between groups (P=0.004) (maintained similar at 12 months)	Mildly Positive:<br><br>Home telehealth provides an innovative and pragmatic approach to enhance earlier detection of key clinical symptoms requiring intervention
**(Wakefield et al. 2012)**<br><br>**US**	Outcomes of a home telehealth intervention for patients with diabetes and hypertension	RCT	High-Intensity Intervention (n=93)<br><br>Low-Intensity intervention (n=102)<br><br>CG (n=107)	Home telehealth device (data transmission and automated responses) and nurse care management (nurse reviewed responses)<br><br>High-Intensity: study team developed branching disease management algorithm (algorithm focused on diet, exercise, smoking, foot care etc.)<br><br>Low-Intensity: No algorithm used<br>CG: usual care; not adequately described	6	• Knowledge scores improved in the high-intensity group participants, but upon further analysis, the intervention effect was not mediated by gain in knowledge<br>• No significant differences were found across the groups in self-efficacy, adherence, or patientperceptions of the intervention mode	Mildly positive:<br><br>Home telehealth can enhance detection of key clinical symptoms that occur between regular physician visits; while intervention improved glycemic and BP control, the mechanism of the effect for this improvement was not clear
**(Luley et al. 2011)**<br><br>**Germany**	Weight loss in obese patients with type 2 diabetes: effects of telemonitoring plus a diet combination – the active body control program	RCT	IG (n=35)<br>CG (n=35)	IG: telemonitoring (homebox sending data to a server) of physical activity + a low calorie diet, weekly reports sent to each patient by mail (with comments and motivation)<br><br>CG: conventional low-fat diet and standard care according to recommendations issued by Deutsche Diabetes Gesellschaft	6	• After 6 months the mean weight loss in IG was 11.8 kg ±8.0 kg (P=0.000), between groups P=0.000<br>• Glucose and HbA1c were lowered by 1.0 mmol/l and 0.8 percentage points (P=0.000 for IG and between groups)<br>• BMI in IG -4.1 (P=0.00 for IG and between groups)<br>• Proportion of patients with HbA1c > 7% fell from 57% to 26%<br>• Antidiabetic drugs were discontinued in 13 patients (39%) and reduced in 14 (42%)<br>• Reduction of costs on medication per patient was €83 in 6 months<br>• In CG no relevant changes in body weight, laboratory values or drug treatment<br>• Blood pressure remained unchanged in both groups	Positive:<br><br>Program effectively lowers body weight, Hb1Ac and antidiabetic drug use

Study	Topic	Design	Participants/ included studies	Intervention/Control	Duration (Months)	Outcomes	Author's conclusions
**(Kim/Kim 2008)**<br><br>**South Korea**	Effectiveness of mobile and internet intervention in obese patients	RCT	IG (n=18)<br>CG (n=16)	IG: web–based intervention; data transmission: weekly diary on website by cellular phone or computer internet; feedback by SMS and internet weekly<br><br>Both: participants in the intervention and the control groups met the endocrinologist specialist 4 or 5 times	12	• HbA1c decreased 1.22% at 3 months, 1.09% at 6 months, 1.47% at 9 months, and 1.49% at 12 months compared with baseline in IG (all time points, P<0.05); percentage change in CG group was not significant<br>• IG had a decrease of 2-h postprandial test of 120.1 mg/dl at 3 months, 58.9 mg/dl at 6 months, 62.0 mg/dl at 9 months, and 102.9 mg/dl at 12 months compared with baseline (all time points, P<0.05); mean change in CG not significant	Positive:<br><br>Improved HbA1c and 2-h postprandial test
**(Bentley et al. 2016)**<br><br>**UK**	Feasibility study of portable technology for weight loss and HbA1c control	RCT	Group 1: no intervention (n=9)<br><br>Group 2: Intervention alone (n=9)<br><br>Group 3: Intervention + support (n=9)	Group 1: no intervention<br><br>Group 2: wearable mHealth device records physical activity, food consumption and provides motivational feedback<br><br>Group 3: as group 2 and additional weekly support via e-mail	4	• Device acceptable, motivational and easy to use by participants<br>• Intervention groups: greater reductions in weight and HbA1c (but small sample size) (no significance reported)	Positive:<br><br>Promising for helping individuals with type 2 diabetes to reduce their HbA1c and lose weight
**(Goodarzi et al. 2012)**<br><br>**Iran**	Impact of distance education via mobile phone text messaging on knowledge, attitude, practice and self efficacy	RCT	IG (n=43)<br>CG (n=38)	IG: 4 messages weekly (SMS)<br><br>CG: no educational messages	3	• IG compared with CG improved significantly in HbA1c (P=0.024), LDL (P=0.019), cholesterol (P=0.002), micro albumin (P≤0.001), knowledge (P≤0.001), practice (P≤0.001) and self efficacy (P≤0.001)	Positive
**(Cho et al. 2009)**<br><br>**Korea**	Mobile communication using a mobile phone with a glucometer - as effective as an internet–based glucose monitoring system	RCT	Phone (n=38)<br>internet (n=37)	Phone group: communication via "diabetes phone"; automatically data transmission to a web server; feedback once every other week by short message service<br><br>Internet group: internet–based glucose monitoring system; manual data transmission to a web server; feedback once every other week via internet	3	• After 3 months, HbA1c levels of both groups decreased significantly, from 7.6% to 6.9% for the internet group and from 8.3% to 7.1% for the phone group (P<0.01)<br>• Mean HbA1c decrement between the two groups was not different significantly (P=0.27)<br>• Levels of patient satisfaction and adherence to medical advice were similar	Positive:<br><br>Mobile phone as effective as internet–based intervention
**(Lim et al. 2016)**<br><br>**Korea**	Multifactorial intervention in diabetes care using real-time monitoring and tailored feedback	RCT	IG (n=50)<br>CG (n=50)	IG: u-health care system; data transmission to main server (home gateway); automatically feedback messages via mobile phone<br><br>CG: SMBG 8 times/ week<br><br>Both: visit u-health care center every 3 months	6	• After 6 months follow-up, HbA1c level was significantly decreased in IG [8.0 ± 0.7% (64.2 ± 8.8 mmol/mol) to 7.3 ± 0.9% (56.7 ± 9.9 mmol/mol)] compared with CG [8.1 ± 0.8% (64.9 ± 9.1 mmol/mol) to 7.9 ± 1.2% (63.2 ± 12.3 mmol/ mol)] (P<0.01)<br>• Proportion of patients with HbA1c <7% without hypoglycemia was greater in IG (26%) than in CG (12%; P<0.05)<br>• Body fat decreased and lipid profiles improved in IG but not CG<br>• BMI significantly decreased in IG (P=0.002)	Positive

Study	Topic	Design	Participants/ included studies	Intervention/Control	Duration (Months)	Outcomes	Author's conclusions
**(Arora et al. 2014)**<br><br>**US**	Text message—based mhealth in emergency department patients with diabetes (TExT-MED)	RCT	IG (n=64)<br>CG (n=64)	IG: 2 daily text messages to mobile telephones (educational, healthy living, medication reminder)<br><br>CG: usual care; not adequately described	6	• HbA1c level decreased by 1.05% in IG compared with 0.60% in controls (∆0.45; 95% CI, –0.27 to 1.17) at 6 months (P>0.05)<br>• Self-reported medication adherence improved from 4.5 to 5.4 in IG compared with a net decrease of –0.1 in controls (∆1.1 [95% CI, 0.1 to 2.1])<br>• Proportion of patients who used emergency services trended lower in IG (35.9% versus 51.6%; ∆15.7%; 95% CI, 9.4% to 22%)	Neutral, positive trend:<br><br>No significant improvement in HbA1c; trends toward improvement in primary outcome of HbA1c and other secondary outcomes
**(Burner et al. 2018)**<br><br>**US**	Using mobile health to improve social support for low-income latino patients with diabetes: mixed-methods analysis of feasibility of TExT-MED + FANS	RCT	Group 1 (n=22)<br>Group 2 (n=22)	Group 1: Patients received the TExTMED program *(see Arora et al. 2014 above)*, their supporters received FANS intervention (1 or 2 text messages daily; FANS = family and friends network supporters)<br><br>Group 2: TExT-med + supporters received a pamphlet mailed at the time of enrollment only with the same information as FANS curriculum with instructions indicating when they should read each message	3	• FANS intervention improved HbA1c (intervention mean decreased from 10.4% to 9.0% vs. from 10.1% to 9.5%, ∆-0.8%, CI -0.4 to 2, P=0.30)<br>• FANS greater drop in mean HbA1c compared with other group, but not significant (P=0.0296)<br>• Self-monitoring of glucose (intervention increased 1.6 days/week vs. control decreased 2 days/week, ∆2.3 days/week, CI 4–0.6, P=0.02)<br>• Physical activity improved 16.1 vs. decreased 9.6 for control, delta 25.7, CI 49.2–2.3, P=0.10)	Positive<br><br>mHealth as feasible, acceptable, and promising avenue to improve social support and outcomes
**(Fang/Deng 2018)**<br><br>**China**	Electronic messaging intervention for management of cardiovascular risk factors	RCT	IG (n=67)<br>CG (n=62)	IG: telemedicine platform (website); monthly recommendations via micro letter + short message on mobile phone; online consulting<br><br>CG: phone calls every 3 months	12	• Statistically significant between-group differences in HbA1c (P=0.034), postprandial plasma glucose (P=0.001), postprandial insulin (P=0.005), total cholesterol (P=0.038), and low-density lipoprotein (P<0.001)<br>• Levels of HbA1c (P=0.011), fasting plasma glucose (P=0.007), postprandial plasma glucose (P＜0.001), fasting insulin (P=0.004), postprandial insulin (P＜0.001), total cholesterol (P＜0.001), and low-density lipoprotein (P＜0.001) decreased significantly in IG<br>• Systolic (P=0.069) and diastolic (P=0.693) BP improvement in IG	Positive
**(Fortmann et al. 2017)**<br><br>**US**	An mHealth SMS–based intervention improves glycemic control in hispanics	RCT	IG (n=63)<br>CG (n=63)	IG: up to 3 motivational, educational, and/or call-to-action text messages per day<br><br>CG: usual care; not adequately described	6	• IG achieved significantly greater reduction in HbA1c over time compared with CG (P=0.03)<br>• Number of blood glucose values texted in by participants was a statistically significant predictor of month 6 HbA1c (P<0.05)	Positive:<br><br>Simple, low-cost text-messaging program was highly acceptable and improved glycemic control

Study	Topic	Design	Participants/ included studies	Intervention/Control	Duration (Months)	Outcomes	Author's conclusions
**(Del Prato et al. 2012)**<br><br>**Italy**	Telecare provides comparable efficacy to conventional self-monitored blood glucose in patients with type 2 diabetes titrating one injection of insulin glulisine—the eleonor study	RCT	IG (n=142)<br>CG (n=149)	Previous: titration phase (8 weeks)<br>IG: data transmission from phone to centralized server from which results are made available to investigator's computer; investigator can then transmit information to the centralized server, which is returned to the patient by phone<br><br>CG: conventional SMBG; diary	6	• After glargine titration, 224 patients achieved FPG ≤7mmol/L, without any difference between groups (mean – SD, 6.2 – 0.8 vs. 6.0 – 0. 9 mmol/L, respectively)<br>• HbA1c levels were lower following titration and were similar for IG and CG (7.9 – 0.9% vs. 7.8 – 0.9% [63 vs. 62 mmol/mol], respectively)<br>• Adding glulisine further reduced HbA1c in both groups (- 0.7% vs. - 0.7%); 45.2% and 54.8% (P=0.14), of patients achieved HbA1c ≤7.0% (≤53 mmol/mol)<br>• No weight change in groups, and hypoglycemia similar between groups	Neutral:<br><br>Patients adding one dose of glulisine at meal with highest postprandial plasma glucose excursion to titrated basal glargine achieved comparable improvements in glycemic control irrespective of traditional or telecare blood glucose monitoring
**(Kim et al. 2016)**<br><br>**China**	Effect of internet–based glucose management system on subjects with diabetes in china	RCT	IG (n=92)<br>CG (n=90)	IG: internet blood glucose monitoring; first 3 months weekly feedback; then biweekly; hospital visits every 3 months<br><br>CG: usual care; hospital visit every 3 months	6	• After 3 months, CG HbA1c levels were reduced from 8.0%±0.8% to 7.3%±1.2% (P<0.001) and IG experienced a reduction from 7.9%±0.8% to 6.9%±0.7% (P<0.001); IG end value was significantly lower (P=0.014)<br>• Intragroup changes were significant at the 3-month (P=0.002) and 6-month (P<0.01) follow-ups<br>• Significant changes in fasting blood sugar were observed in IG at month 3 (P=0.003) and month 6 (P=0.005)	Positive
**(Hanley et al. 2015)**<br><br>**Scotland and England**	Qualitative study of telemonitoring of blood glucose and BP in type 2 diabetes	Qualitative	Patients (n=23)<br>Nurses (n=6)<br>Doctors (n=4)<br><br>Semi-structured interviews	IG: Telemonitoring: data transmission via modem/web–based; monthly feedback via post or e-mail<br><br>CG: usual care; visit nurse or doctor up to 3-4 times (average)	6	• Motivation to self-manage diet was increased by telemonitoring of blood glucose<br>• The 'benign policing' aspect of telemonitoring was considered by patients to be important<br>• Convenience of home monitoring was very acceptable to patients although professionals had some concerns about telemonitoring increasing workload and costs	Positive:<br><br>Telemonitoring of blood glucose, BP and weight in primary care is a promising way of improving diabetes management

Study	Topic	Design	Participants/ included studies	Intervention/Control	Duration (Months)	Outcomes	Author's conclusions
**(Lee et al. 2019)**<br><br>**Malaysia**	Using telemedicine to support care for people with type 2 diabetes mellitus: qualitative analysis of patients' perspectives	Qualitative	Patients (n=48) who partici-pated in a RCT<br><br>In-depth and focus group interviews	*See Lee et al. 2020*		• Main obstacles found in patients using the telemedicine systems were related to internet connectivity and difficulties experienced with system interface<br>• Cost was also another significant concern raised by participants<br>• Participants were primarily positive about the benefits of telemedicine, including its ability to provide real-time data and disease monitoring and the reduction in clinic visits	Positive:<br><br>Collaboration between educators, health care providers, telecommunication service providers and patients are required to stimulate the adoption and the use of telemedicine
**"Combined interventions" (real-time and asynchronous communication) (n=30)**							
**(Parsons et al. 2019)**<br><br>**UK**	Effect of structured self-monitoring of blood glucose, with and without additional TeleCare support, on overall glycaemic control in non-insulin treated type 2 diabetes	RCT	Group 1: usual care (CG) (n=151)<br><br>Group 2: SMBG (n=147)<br><br>Group 3: SMBG + TeleCare (n=148)	Group 1: usual care, not adequately described<br><br>Group 2: SMBG<br><br>Group 3: monthly TeleCare support; data transmission via securely e-mail or phone; feedback via monthly phone call	12	• Compared to baseline, mean HbA1c was lower in all groups at 12months, with reductions of 3.3mmol/mol (95%CI –5.71 to –0.78) or 0.3% (95%CI –0.52 to –0.07; P=0.01) in CG, 11.4mmol/mol (95%CI –14.11 to –8.76) or 1.1% (–1.29 to –0.81; P<0.0001) in the group using SMBG alone and 12.8 mmol/mol (95%CI –15.34 to –10.31) or 1.2% (95%CI –1.40 to -0.94; P<0.0001) in the group using SMBG + TeleCare<br>• At 12 months, greater reduction mean HbA1c in the combined SMBG group of 12.2 mmol/mol (1.1%) compared to 3.3 mmol/mol (0.3%) in CG, with the difference being significant, after adjusting for age at screening, gender, total cholesterol, BMI and duration of diabetes (P<0.0001)	Neutral:<br><br>No additional benefit, over and above the use of structured SMBG, was observed in glycaemic control with the addition of monthly TeleCare support

Study	Topic	Design	Participants/ included studies	Intervention/Control	Duration (Months)	Outcomes	Author's conclusions
**(Carter et al. 2011)**<br><br>**US**	Patient-centric, provider-assisted diabetes telehealth self-management intervention for urban minorities	RCT	IG (n=26)<br>CG (n=21)	IG: 3 modules: self-management (biweekly 30-minute video-conferences), health education (videos, websites, material on nutrition, physical activity, weight loss etc.), social networking (linking all participants)<br><br>CG: usual care; not adequately described	9	• Significant association between participation in intervention and achieving a HbA1c measure of 7 percent or below, P<0.05<br>• Significant positive relationship between participation in the intervention and achieving a healthy BMI, P<0.05<br>• No such association was found between being in the treatment group and maintaining blood pressure at 130/80, P>0.05<br>• IG reported increased knowledge of diabetes and improved adherence to sound diabetes management practices such as regular feet checks, P<0.05<br>• IG feeling better mentally and physically, P<0.05	Positive:<br><br>Findings support the development of telehealth interventions to promote effective chronic disease management in medically underserved communities
**(Bujnowska-Fedak et al. 2011)**<br><br>P**oland**	Impact of telehome care on health status and QoL among patients with diabetes in a primary care setting in poland	RCT	IG (n=50)<br>CG (n=50)	IG: patient unit and medical (or provider) unit, connected by a computer network; data transmission via internet; feedback via e-mail, urgent cases via phone call<br><br>CG: usual care; not adequately described	6	• Overall reduction in HbA1c values in both groups after 6 months<br>• Significant difference in HbA1c values between the groups was observed only among the noninsulin-requiring patients (decline from 6.95% – 0.82% to 6.66% – 0.86% in IB vs. 7.21% – 2.02% to 7.2% – 1.86% in IIB; P=0.02)<br>• IG reported considerably less hyperglycemic and hypoglycemic events<br>• IG had higher overall scores on diabetes related QoL measures (not significant between groups)<br>• Positive association between educational attainment and ability to use telemonitoring system without help (P=0.045)	Not conclusive, but positive trend:<br><br>Because of the small sample and short observation period
**(Jeong et al. 2018)**<br><br>**South Korea**	Smart care –based on telemonitoring and telemedicine for type 2 Diabetes care	RCT	Telemedicine (n=112)<br>Telemonitoring (n=113)<br>CG (n=113)	Telemedicine group: video-conferencing (web-enabled computer) with endocrinologist twice; automated short message feedback + SMBG + 1 face-to-face outpatient visit<br><br>Telemonitoring group: assessment by 3 outpatient visits + SMBG + automated short message<br><br>CG: 4 outpatient appointments + SMBG	6	• Reductions in HbA1c concentration after 24 weeks were similar in all groups (-0.66% – 1.03% vs. -0.66% – 1.09% vs. -0.81% – 1.05%; P>0.05 for each pairwise comparison, P<0.0001 for within-group difference)<br>• Fasting blood glucose concentrations were significantly lower in the telemonitoring (P=0.0247) and telemedicine (P=0.0266) groups than in CG<br>• Rates of hypoglycemia were significantly lower in the telemedicine group than in the other two groups (P<0.05)<br>• Compliance with medication was significantly better in telemonitoring (P=0.0471) and telemedicine (P=0.0001) than in CG<br>• No serious adverse events	Neutral:<br><br>Telehealth care was as effective as conventional care at improving glycemia in patients with type 2 diabetes without serious adverse effects

Study	Topic	Design	Participants/ included studies	Intervention/Control	Duration (Months)	Outcomes	Author's conclusions
**(Rodriguez-Idigoras et al. 2009)**<br><br>**Spain**	Telemedicine influence on the follow-up of type 2 diabetes patients	RCT	IG (n=161)<br>CG (n=167)	IG: data transmission to call center via mobile phone; physicians feedback via mobile phone and phone calls<br><br>CG: not adequately described	12	• Reduction in HbA1c after 12 months from 7.62±1.60% to 7.40±1.43% (P=0.027) in IG; from 7.44±1.31% to 7.35±1.38% (P=0.303) in CG<br>• Difference in change between groups not statistically significant at 12-months follow up (P=0.342)<br>• Significant decrease in BP (systolic P=0.003, diastolic P=0.025), total cholesterol (P=0.015), low-density lipoprotein cholesterol (P=0.016), and BMI (P=0.047) in IG<br>• In CG, only significant decline was in low-density lipoprotein cholesterol	Positive
**(Kempf et al. 2017)**<br><br>**Germany**	Telemedical Lifestyle intervention program Telipro in advanced stages of type 2 diabetes	RCT	IG (n=102)<br>CG (n=100)	IG: TeLiPro program: telemedical coaching incl. medical-mental motivation, formula diet, SMBG; data transmission via online portal; weekly care calls<br><br>CG: quarterly visits with physician	3	• After 3 months: HbA1c reduction was significantly higher in TeLiPro (mean ± SD -1.1 ± 1.2% vs. -0.2 ± 0.8%; P<0.0001)<br>• Treatment superiority of TeLiPro was maintained during follow-up (week 26: 0.6% [95% CI 1.0; 0.3], P=0.0001; week 52: 0.6% [0.9; 0.2], P<0.001)<br>• Same applies for weight (TeLiPro -6.2 ± 4.6 kg vs. control -1.0 ± 3.4 kg), BMI (-2.1 ± 1.5 kg/m² vs. -0.3 ± 1.1 kg/m²), systolic BP (-5.7 ± 15.3 mmHg vs. -1.6 ± 13.8mmHg), 10-year cardiovascular disease risk, antidiabetes-medication, HRQoL and eating behavior (P<0.01 for all)<br>• Effects were maintained longterm<br>• No adverse events	Positive:<br><br>In advanced-stage type 2 diabetes, TeLiPro can improve glycemic control and may offer new options to avoid pharmacological intensification
**(Wang et al. 2017)**<br><br>**China**	Application of telemedicine in the management of type 2 diabetes mellitus	RCT	IG (n=106)<br>CG (n=106)	IG: internet–based; u-health care website for data transmission; feedback by medical team every 2 weeks via website or telephone<br><br>CG: glucometers without any other requirement<br><br>Both: clinic visits every 3 months	6	• At the 3-month follow-up, HbA1c and fasting plasma glucose levels of IG were significantly lower than those at the baseline (P=0.000 and P=0.001) as well as those of CG (P=0.003 and P=0.038)<br>• Triglyceride levels of the IG were much lower than those at the baseline<br>• At the 6-month follow-up, 2-hour postprandial plasma glucose levels of IG significantly improved compared with those of CG<br>• HbA1c levels gradually decreased every 3 months in IG, and the mean change in the levels was significantly greater in this group than in CG (from 1.27% to 0.68%)	Positive:<br><br>internet–based u-health care system of integrated management in diabetes not only achieved better glycemic control, effectively improved HbA1c levels, and decreased triglyceride levels but also enhanced patients' adherence to the medical team's instructions

Study	Topic	Design	Participants/ included studies	Intervention/Control	Duration (Months)	Outcomes	Author's conclusions
**(Zhou et al. 2014)**<br><br>**China**	Web–based telemedicine for management of type 2 diabetes through glucose uploads	RCT	IG (n=57)<br>CG (n=57)	IG: internet–based system; data transmission every 2 weeks; feedback through internet, short messages or telephone<br><br>CG: individual face-to-face-visits	3	• Compared to CG, IG exhibited better HbA1c and fasting blood glucose controlling (P<0.05), also significant improvement within IG compared to baseline (P<0.001)<br>• Telemedicine decreased hypoglycemia risk (P=0.044), and contributed to levels of HbA1c less than 7% which is the target of our study (P=0.049)<br>• IG: BMI no effect (P=0.987)<br>• IG: systolic and diastolic BP improved (P=0.004 and 0.005)	Positive:<br><br>Telemedicine system can provide a tighter glycemic control for the treatment of type 2 diabetes patients, especially in cases with difficulties to access to the medical centre
**(Chen et al. 2011)**<br><br>**Taiwan**	Efficacy and safety of the telehealth system in poorly controlled type 2 diabetic patients receiving insulin therapy	RCT	IG (n=32)<br>CG (n=32)	IG: education program (clinic visits every 3 months; 4 phone calls) + telehealth data analysis platform (for data transmission) + telephone system<br><br>CG: usual care, not adequately described	12	• IG: significantly improved HbA1c levels (9.5% ± 1.8% to 8.1% ± 1.2%; P<0.01) without significant body weight change (P=0.231)<br>• CG: no significant improvement in HbA1c levels but significant increase in body weight (66.8 ± 9.8 to 67.3 ± 10.0 kg; P<0.01)<br>• No patient in IG was hospitalized during follow-up period, but 6 patients in CG<br>• Intergroup differences in hypoglycemic events were absent	Positive:<br><br>Useful education method to improve blood sugar control and prevent hospitalization
**(Nicolucci et al. 2015)**<br><br>**Italy**	Home telemonitoring for the management of metabolic and cardiovascular risk in patients with type 2 diabetes	RCT	IG (n=153)<br>CG (n=149)	IG: home telehealth system (individual messages by text message, e-mail or telephone); nurses contacting patients monthly by phone<br><br>CG: usual care; not adequately described	12	• Use of the telemonitoring associated with a statistically significant reduction in HbA1c levels compared with CG (estimated mean difference, 0.33 – 0.1; P=0.001)<br>• Improvements in body weight (P=0.66) BP, systolic BP (P=0.58) and diastolic BP (P=0.62), but not significant compared with CG<br>• No differences between groups for lipid profile (all P>0.05)<br>• As for HRQoL, significant differences in favor of IG were detected as for physical functioning (P=0.01), role limitations due to emotional problems (P = 0.02), mental health (P = 0.005), and mental component summary (P = 0.03) scores<br>• Lower number of specialist visits in IG (incidence rate ratio, 0.72; 95% CI, 0.51–1.01; P=0.06)	Mildly Positive:<br><br>Better metabolic control and QoL<br>No impact on BP, lipid profile, and body weight

Study	Topic	Design	Participants/ included studies	Intervention/Control	Duration (Months)	Outcomes	Author's conclusions
**(Pressman et al. 2014)**<br><br>**US**	A novel telemonitoring device for improving diabetes control	RCT	IG (n=118)<br>CG (n=107)	IG: telemetry device (installed at home) for weekly data transmission; care managers individually responded via telephone or other method, including telemetry device + send educational information via telemetry device<br><br>CG:usual care; not adequately descibed	6	• At 6 months, systolic BP -3.2 mmHg in IG (P=0.483 compared to CG)<br>• HbA1c improved significantly over 6 months in both groups (P-value not reported)<br>• At 6 months, no significant intergroup differences in change from baseline for HbA1c (-2.0% in IG, P= 0.310), fructosamine (P=0.881), or self-efficacy (P=0.319)<br>• LDL cholesterol in IG decreased more than in CG (-17.1mg/dL versus -5.4mg/dL; P=0.045)<br>• BMI mean -0.2 kg/m² in IG (P=0.804 compared to CG)<br>• Weight mean -0.1 pounds in IG (P=0.677 compared to CG)	None:<br><br>Although HbA1c improved significantly over 6 months in both groups, the difference in improvement between the groups was not significant<br><br>This lack of significance may be due to the relatively healthy status of the volunteers and to the high level of care provided by the care managers
**(Stone et al. 2010)**<br><br>**US**	Active care management supported by home telemonitoring in veterans with type 2 diabetes (Diatel)	RCT	Group 1 (n=73)<br>Group 2 (n=77)	Group 1: Active care management with home telemonitoring: 2-h educational session; data transmission via telemonitoring device (SMBG and other measurements, messaging with reminders and education); monthly counseling by nurse via telephone<br><br>Group 2: 2-h educational session; monthly care coordination telephone call	6	• Compared with the telephone group, the telemonitoring group demonstrated significantly larger decreases in A1c at 3 months (1.7 vs. 0.7%) and 6 months (1.7 vs. 0.8%; P<0.001 for each), with most improvement occurring by 3 months<br>• Weight increase in IG at 6 months compared to CG (P=0.49)<br>• Systolic and diastolic BP decreased at 6 months in IG compared to CG (P>0.05)<br>• None of the other primary outcomes differed significantly by treatment group at either 3 or 6 months, but direction of the differences favored IG (cholesterol, LDL, triglycerides)	Positive:<br><br>Both interventions improved glycemic control; telemonitoring group significantly greater reductions in A1c by 3 and 6 months

Study	Topic	Design	Participants/ included studies	Intervention/Control	Duration (Months)	Outcomes	Author's conclusions
**(Stone et al. 2012)**<br><br>**US**	Diabetes telemonitoring study extension - alternative interventions to maintain glycemic control after withdrawal of diabetes home telemonitoring	RCT	ACM-to-CCHT (n=23)<br>ACM-to-CC (n=21)<br><br>CC-to-CC (n=28)<br>CC-to-UC (n=29)	Participations receiving active care management *(see Stone et al. 2010)* re-assigned:<br><br>ACM-to-CCHT: monthly care coordination calls with telemonitoring but no active medication management<br><br>ACM-to-CC: monthly care coordination telephone calls<br><br>Particication receiving care coordination re-assigned:<br><br>CC-to-CC: continued care coordination<br><br>CC-to-UC: usual care	6	• Marked HbA1c improvements observed in DiaTel ACM participants were sustained 6 months after re-randomization in both ACM-to-CCHT and ACM-to-CC groups<br>• No benefit was apparent for continued transmission of glucose data among DiaTel ACM participants or continued monthly telephone calls among DiaTel CC participants 6 months after re-randomization<br>• Only within-group change that approached statistical significance was an increase in HbA1c of 0.35% in the CC-to-UC group between 6 and 9 months (P=0.06; P>0.20 for all other within-group comparisons over time)<br>• largest, although not significant, pairwise differences were between the ACM-to-CC and CC-to-CC groups (0.59% at 6 months; P=0.11, reflecting DiaTel differences); 0.49% at 9 months, P=0.19; and 0.55% at 12 months, P=0.11<br>• None of the remaining pairwise differences approached statistical significance at 6, 9, or 12 months (P>0.30 for each)	Positive:<br><br>Significant improvements in HbA1c achieved using home telemonitoring and active medication management for 6 months were sustained 6 months later with interventions of decreased intensity
**(Hsu et al. 2016)**<br><br>**US**	Utilization of a cloud–based diabetes management program for insulin initiation and titration	RCT	IG (n=20)<br>CG (n=20)	IG: cloud–based diabetes management program (self-tracking tools, secure text messages, virtual visits via audio, video and shared screen control)<br><br>CG: standard face-to-face care and phone follow up when needed	3	• IG achieved a greater HbA1c decline compared with the control group (3.2 – 1.5% vs. 2.0% – 2.0%; P=0.048)<br>• At the end of the study, IG achieved a mean HbA1c decrease of 3.2 – 1.5% (P < 0.0001)<br>• No significant changes in weight (–0.48 pound in the intervention group vs. -0.87 pound in the control group; P=0.9)<br>• Mean virtual visit time of 22.5 min per subject, whereas the control group had a mean of 68.8 min for visit time with clinicians<br>• Diabetes Treatment Satisfaction Questionnaire showed significant improvement in IG compared with CG (increase of 10.1 – 11.7 vs. 2.1 – 6.5 points; P=0.01)	Positive:<br><br>Mobile health technology could be an effective tool in sharing data, enhancing communication, and improving glycemic control
**(Wakefield et al. 2014)**<br><br>**US**	Effect of home telemonitoring on glycemic and blood pressure control in primary care clinic patients	RCT	IG (n=55)<br>CG (n=53)	IG: "Connectivity Hub" (daily data transsmission via analog phone line or personal computer with internet access); feedback via telephone call<br><br>CG: diary + clinic visits	3	• No significant differences between groups on either A1c or systolic BP following the intervention<br>• On average, participants' A1c worsened slightly over time; rate slower in IG (0.03) than CG (0.05) after 180 days (Significance not reported)<br>• SBP improved slightly over time for everyone (Significance not reported)	None:<br><br>Addition of technology alone is unlikely to lead to improvements in outcomes

Study	Topic	Design	Participants/ included studies	Intervention/Control	Duration (Months)	Outcomes	Author's conclusions
**(Crowley et al. 2016)**<br><br>**US**	Practical telemedicine for veterans with persistently poor diabetes control	RCT, pilot	IG (n=25)<br>CG (n=25)	IG: telemonitoring („Teleresponse" interactive voice response system) SMBG; telephonic feedback every 2 weeks + diabetes self-management support module + physician-guided medication and depression management<br><br>CG: usual care + educational packet	6	• By 6 months, HbA1c had improved by 1.3% for IG and 0.3% for CG (estimated difference, -1.0%, 95% CI, -2.0%, 0.0%; P=0.050)<br>• IG: diabetes self-care (estimated difference, 7.0; 95%CI, 0.1, 14.0; P=0.047), systolic BP (-7.7mm Hg; 95% CI, -14.8, -0.6; P=0.035), and diastolic BP (–5.6mm Hg; 95% CI, -9.9, -1.2; P=0.013) were improved versus CG by 6 months	Positive
**(Warren et al. 2018)**<br><br>**Australia**	Effects of telemonitoring on glycaemic control and health care costs in type 2 diabetes	RCT	IG (n=63)<br>CG (n=63)	Both: 3 home visits<br><br>IG: usual care + telehealth: home telehealth devices (tablet computer for videoconferencing, educational videos etc.); data transmission via internet; response –based on individual need<br><br>CG: usual care; not adequately described	6	• HbA1c in IG decreased from a median 68 mmol/mol (8.4%) to 58 mmol/mol (7.5%), and remained unchanged in CG at median 65 mmol/mol (8.1%) at the 6-month endpoint<br>• HbA1c values in IG over time was significantly greater than in CG (P<0.01)<br>• Intervention effect on HbA1c change was statistically signicant (P=0.004)<br>• Total health care costs in IG, including the intervention costs, were lower (mean $3781 vs. $4662; P<0.001) compared with CG	Positive:<br><br>Clinically meaningful and statistically significant benefit from the telehealth intervention at a lower cost
**(Castelnuovo et al. 2011)**<br><br>**Italy**	TECNOB study: ad interim results of a randomized controlled trial of a multidisciplinary telecare intervention for obese patients	RCT	IG (n=17)<br>CG (n=17)	Both: 1-month inpatient intensive program that involved medical care, diet therapy, physical training and brief psychological counseling<br><br>IG: weight-loss website, web–based videoconference tool, dietary software on cellular phones and electronic armband<br><br>CG: did not receive any instructions and were discharged as other inpatients not included in the study	12	• No statistically significant difference between groups in weight change at any time-point<br>• Within IG group: significant reductions of initial weight at discharge (-5,9 kg, P<0.000), at 3 months (-8,2 kg, P<0.000) and at 6 months (-7,6 kg, P=0.002); reduction of baseline weight at 12 months not statistically significant (-6,9 kg, P=0.136)<br>• Control group had higher scores in Interpersonal distrust at 12 months	None:<br><br>Effect of the inpatient treatment was high and probably overwhelmed the effect of the TECNOB intervention
**(von Storch et al. 2019)**<br><br>**Germany**	Telemedicine-assisted self-management program	RCT	IG (n=60)<br>CG (n=55)	IG: telemonitoring with lifestyle intervention through individual need–based telephone coaching; data transmission and communication via tablet computer<br><br>CG: usual care; not adequately described	3	• IG resulted in significantly greater declines in HbA1c compared with CG (-0.463%); P=0.038<br>• IG showed significant improvements in diabetes self-management scale score (+0.903 points; P=0.000) and BMI (-0.566 kg/m²; P=0.036) compared with CG<br>• Within-IG improvements of these outcomes are also significant (all P=0.000)	Positive
**(Liou et al. 2014)**<br><br>**Taiwan**	Shared care combined with telecare improves glycemic control of diabetic patients in a rural underserved community	RCT	IG (n=54)<br>CG (n=41)	IG: shared care combined with telecare; 6 sessions (1 introduction; 3 educational in-person internet sessions; 2 video-conferencing with care team (nurse, dietitian, diabetic specialist)<br><br>CG: 1 education session by nurse	6	• Decrease in HbA1c level was significantly greater in IG than in CG (0.7 – 1.3% versus 0.1 – 1.0%, P=0.03)<br>• Improvements in BMI, SBP, DBP, cholesterol, LDL in IG, but not significant between groups	Positive:<br><br>Shared care combined with telecare could significantly reduce HbA1c levels in type 2 diabetic patients with poor glycemic control in underserved rural communities

Study	Topic	Design	Participants/ included studies	Intervention/Control	Duration (Months)	Outcomes	Author's conclusions
**(Plotnikoff et al. 2013)**<br><br>**Canada**	The alberta diabetes and physical activity trial (adapt): theory–based interventions to increase physical activity in adults with type 2 diabetes	RCT	Group 1 (n=94)<br>Group 2 (n=97)<br>Group 3 (n=96)	Group 1: control (standard print educational materials by Canadian Diabetes Association)<br><br>Group 2: print–based materials/ pedometer (tailored, specific educational materials mailed every 3 months)<br><br>Group 3: print–based materials/ pedometer like group 1 and 2 + telephone-counseling (first month weekly calls, next month biweekly calls, and for remainder of the intervention calls were monthly)	12	• Physical activity and HbA1c levels did not significantly change in intervention groups<br>• Step counts significantly increased in group 2, for women<br>• HbA1c at 12 months: group 3 (7.11% to 7.28%) vs. group 1 (7.08% to 7.07%), P=0.153<br>• Fasting glucose at 12 months group 3 (8.0 mmol/l to 8.0 mmol/l) vs. group 1 (7.8 mmol/l to 7.8 mmol/l), P=1.0<br>• BMI at 12 months: group 3 (30.2 kg/m² to 30.5 kg/m²) vs. group 1 (30.2 kg/m² to 30.5 kg/m²), P=0.965	Mildly positive:<br><br>Multi-component strategy including telephone counseling may have potential for women
**(McFarland et al. 2012)**<br><br>**US**	Use of home telehealth monitoring with active medication therapy management by clinical pharmacists	Non-randomized, controlled clinical trial	IG (n=36)<br>CG (n=67)	IG: 3 clinic visits; data transmission via messaging device in the time intervals between face-to-face visits; feedback (clinical pharmacy and nurse) specialist via telephone<br><br>CG: followed between face-to-face visits by telephone calls	6	• A statistically significant difference in mean HbA1c was noted in IG vs. CG at 3 months (7.2% vs 8.0%, P=0.0002) and 6 months (6.9% vs 7.5%, P=0.0066)<br>• Mean reduction in HbA1c from baseline to 6 months was not significant between groups (P=0.1987)<br>• 69% of IG versus 36% in CG achieved the American Diabates Association HbA1c goal of less than 7% (P=0.0011)	Mildly positive
**(Kesavadev et al. 2012)**<br><br>**India**	Cost-Effective Use of Telemedicine and SMBG via Diabetes Tele Management System (DTMS) to Achieve Target Glycosylated Hemoglobin Values	Retro-spective cohort study	n=1000	DTMS: SMBG + three telemedicine follow-up options to the patients: via phone, e-mail, and secure website; individual advice from team (physicians, diabetes educators, dieticians, nurses, pharmacists, and psychologists) through DTMS (education, counseling) + patients are invited to educational seminars of 4–5h in duration once every 2 months	6	• Patients showed a significant reduction in HbA1c from baseline at months 3 and 6 of 2.2% (P<0.0001)<br>• Recurring extra cost to patient for DTMS, not considering cost of oral drugs and insulin, was equivalent to 9.66 U.S. dollars/month (significance not reported), but money and time saved in physical visits make up for the extra costs<br>• FBG (-67 mg/dL), BMI (-0.3 kg/m²), BP (systolic -9.6 mm Hg, diastolic -4.5 mm Hg), serum creatinine (-0.06 mg/dL), LDL (-44 mg/dL), HDL (+3 mg/dL), triglycerides (-35 mg/dL) and total cholesterol (-56 mg/dL) all improved significantly (all P<0.01)	Positive:<br><br>DTMS appears to be safe and cost-effective in the intensive treatment of T2D without serious co-morbidities
**(Jia et al. 2009)**<br><br>**US**	Health service utilization for diabetes patients in a care coordination home-telehealth programme	Retro-spective cohort study	IG (n=387)<br>CG (n=387)	IG: daily monitoring via messaging device connected to telephone socket; feedback: telephone call (medication management); in rare circumstances, a telemonitor and a videophone were used for weekly contact<br><br>CG: not adequately described	48	• Compared with the controls, IG were less likely to be admitted for inpatient care during the first (P=0.001) and second (P=0.01) six-month follow-up period, and were consistently more likely to visit outpatient clinics (P=0.001) during the whole 48-month follow-up period<br>• The likelihood of increase in outpatient utilization tended to decline over time	Positive:<br><br>Programme helped to reduce overall inpatient and outpatient use by the clients

Study	Topic	Design	Participants/ included studies	Intervention/Control	Duration (Months)	Outcomes	Author's conclusions
**(Jha et al. 2016)**<br><br>**India**	Effectiveness of an electronic health and mobile health platform versus conventional care	Prospective obser-vational study	IG (n=109)<br>CG (n=70)	IG: scheduled visits + weekly telephonic follow-up by team physicians/diabetes educators + educational videos + daily tips via e-mail or text message<br><br>CG: scheduled visits	approx. 8 months	• Statistically significant reduction in HbA1c (8.8 ± 1.2 to 7.4 ± 1.3, P=0.001) in IG post intervention<br>• Fasting blood sugars/ 2-h postprandial blood sugars showed trend towards improvement in both arms, but intergroup correlation at study end not statistical significant (P=0.5); fasting blood sugars improved significantly within IG (177.3 ± 74.3 mg/dL to 119 ± 21.5 mg/dL, P=0.000)<br>• Diabetes knowledge scores (19.9 ± 2.5 vs. 17.9 ± 3.98, P=0.005) and DRQoL indices (88.5 ± 7.8 vs. 83.5 ± 10.7, P=0.015) showed a statistically significant improvement in IG	Positive
**(Lewinski et al. 2019)**<br><br>**US**	Addressing diabetes and poorly controlled hypertension: pragmatic mhealth self-management intervention	Non-controlled intervention study	n=141	Telephone–based intervention: monthly telephone calls; monthly e-mails; weekly text messages (education,medication adherence and self-management)	6	• Proportion of participants with poor baseline SBP control (50/118, 42.4%) did not change significantly (53/118, 44.9%) (P=0.64)<br>• Participants who completed 4 or more phone calls (98/118, 83.1%) did not experience statistically significant decrease in SBP when compared to those who completed fewer calls	None:<br><br>No reduction in uncontrolled hypertension
**(Koopman et al. 2014)**<br><br>**US**	Implementing home blood glucose and blood pressure telemonitoring in primary care practices for patients with diabetes	Qualitative	Nurse care coordinators (n=6) (Semi-structured interviews)<br><br>Physicians (n=12) (Semi-structured interviews)<br><br>Patients (n=93) (Exit-Interviews)	IG: grounded theory qualitative analysis in parallel with a RCT of home telemonitoring (website, feedback twice weekly via web or telephone call)<br><br>CG: written values to clinic visits	12	• (1) understand the capabilities and limitations of the technology and the willingness of patient and physician stakeholders to use it, (2) understand the workflow, flow of information, and human factors needed to optimize use of the technology, (3) engage and prepare the physicians, and (4) involve the patient in the process<br>• Enthusiasm for a patient-centered medical home model that included between-visit telemonitoring, there was concern about the support and resources needed to provide this service to patients	Tends to be positive:<br><br>Careful consideration of workflow and information flow will help enable effective implementations
**(Andrews et al. 2017)**<br><br>**US**	Patient perceptions of a comprehensive telemedicine intervention	Qualitative	Veterans (n=18)<br><br>Semi-structured interviews	Daily monitoring via TeleResponse Interactive Voice Response System; feedback via phone call	6	• Greater awareness of their blood glucose levels<br>• Dissatisfaction with telemonitoring interface and competing demands during intervention<br>• Participants with <1% HbA1c improvement reported that these challenges interfered with their engagement<br>• Participants with ≥1% HbA1c improvement reported new self-management routines despite challenges	Mildly positive:<br><br>Future work should focus on optimizing systems' telemedicine infrastructure

Study	Topic	Design	Participants/ included studies	Intervention/Control	Duration (Months)	Outcomes	Author's conclusions
**(Lee et al. 2018)**<br><br>**UK**	Patients' perception of using telehealth	Qualitative	Patients (n=10)<br><br>Semi-stuctured interviews	Participants from the "whole system demonstrator programme" (RCT of telehealth and telecare - diabetes, heart failure and chronic obstructive pulmonary disease)<br><br>Home monitoring system (base unit); data transmission via this system and feedback via system or telephone	18-42	• Pleased with the technology and many also proclaimed that they could not see themselves being without it<br>• Very few negative views<br>• Telehealth is only viewed as an additional health service to patients' regular care	Positive
**(Dienstl et al. 2011)**<br><br>**Germany**	Effect of telemedicine on glucometabolic control and QoL in patients with type 2 diabetes mellitus	Obser-vational study	Telemedical program (n=100)	"Home Care Communication Center" data transmission via modem; feedback when needed via telephone	6	• Participation in the telemedical program was associated with a significant improvement of HbA1c (–0.4%) as well as cardiovascular risk factors, i. e. weight (-1.3 kg), BMI (-0.7 kg/m²), fasting blood glucose (-26.5 mg/dL) and BP (systolic -9.1 mm Hg, diastolic – 4.8 mm Hg), all P<0.0001<br>• Significant decrease of diabetes specific impairment and increase in DRQoL (P<0.001)<br>• The higher the HbA1c at baseline, the larger their diabetes specific impairment (r=0.299; P=0.007)<br>• Number of depressive patients decreased after 3 months from 64 to 35 (P<0.0001)	Mildly Positive:<br><br>Telemedical care is interesting for those patients, for whom conventional diabetes care is not sufficient to assure good metabolic control
**"Combined interventions – subgroup video clips" (n=3)**							
**(Tang et al. 2013)**<br><br>**US**	Online disease management of diabetes: engaging and motivating patients online with enhanced resources-diabetes	RCT	IG (n=193)<br>CG (n=193)	IG: internet–based, text and video education; individual frequency; delivered by nurse case managers through the personal health records<br><br>CG: usual care; not adequately described	12	• IG had significantly reduced HbA1c at 6 months (−1.32% IG vs −0.66% CC; P<0.001)<br>• At 12 months, the differences were not significant (−1.14% IG vs −0.95% CG; P=0.133)<br>• Significantly more IG patients had improved diabetes control (>0.5% reduction in A1c) than CG patients at 12 months (69.9 (95% CI 63.2 to 76.5) vs 55.4 (95% CI 48.4 to 62.5); P=0.006)<br>• IG significantly better control of LDL compared to CG (P=0.001) at 12 months<br>• No weight change in both groups (P=0.232) at 12 months<br>• IG improvements in systolic and diastolic BP, but not significant at any timepoint compared to CG (12 months: systolic P=0.306 and diastolic P=0.374)	Mildly positive:<br><br>Differences were not sustained at 12 months

Study	Topic	Design	Participants/ included studies	Intervention/Control	Duration (Months)	Outcomes	Author's conclusions
**(Greenwood et al. 2015)**<br><br>**US**	Overcoming Clinical Inertia: randomized clinical trial of a telehealth remote monitoring intervention using paired glucose testing in adults with type 2 diabetes	RCT	IG(n=45)<br>CG (n=45)	IG: in-home tablet computer connected by internet (data transmission); 84 sequential daily health sessions (videos, PowerPoint; feedback: asynchronous virtual visits; after SMBG analysis, diabetes educators generated a virtual visit via asynchronous secure messaging<br><br>CG: diabetes education booklets and referral for formal diabetes education as needed; telephone/ secure messaging feedback	6	• Both groups lowered A1c with an estimated average decrease of 0.70% in CG and 1.11% in IG with a significant difference of 0.41% at 6 months (SE 0.08, P=0.005)<br>• Change in medication (SE 0.21, P=0.009) was significantly associated with lower A1c level<br>• IG significantly improved on the self-reported Summary of Diabetes Self-Care Activities subscales carbohydrate spacing (P=0.04), monitoring glucose (P=0.001), and foot care (P=0.02)	Positive:<br><br>eHealth model significantly improved A1c levels compared to usual care
**(Steventon et al. 2014)**<br><br>**UK**	Effect of telehealth on glycaemic control	RCT	IG (n=488)<br>CG (n=379)	IG: Usual care + telehealth (telemonitoring, educational messages/videos via telehealth base unit or set top box connected to a television; individual frequency)<br><br>CG: SMBG; usual care not adequately described	12	• Telehealth was associated with lower HbA1c than usual care during the trial period (difference 0.21% or 2.3 mmol/mol, 95% CI, 0.04% to 0.38%, P=0.013)<br>• Among the 457 patients in the secondary analysis, mean HbA1c showed little change for controls following recruitment, but fell for intervention patients from 8.38% to 8.15% (68 to 66 mmol/mol)	Mildly Positive:<br><br>Telehealth modestly improved glycaemic control in type 2 diabetes patients over 12 months

BP = blood pressure; CC = couple calls; CG = control group; CI = confidence interval, DE = diabetes education; DRQoL = diabetes-related quality of life; eHealth = electronic Health; DTMS = diabetes tele management system; FBG = fasting blood glucose; eHealth = electronic health; GDM = gestational diabetes mellitus; HbA1c = hemoglobin A1c; HRQoL = health-related quality of life; ID = individual calls; IBGMS = internet blood glucose monitoring system; IG = intervention group; IRT = intensive remote monitoring; MA = meta-analysis; MD = mean deviation; mHealth = mobile health; N/S = Not significant; OR = odds ratio; QoL = quality of life; RCT = randomized controlled trial;; RT-CGM = real-time continuous glucose monitoring; SD = standard deviation; SE = standard error; SMBG = self monitoring blood glucose; SMS = short message service; SR = systematic review; T2DM = type 2 diabetes mellitus, WMD = weighted mean difference.
