# Supplementary material for: Effect of Telemetric Interventions on Glycated Hemoglobin A1c and Management of Type 2 Diabetes Mellitus: Systematic Meta-Review
Source: J Med Internet Res. 2021 Feb 17;23(2):e23252. doi: 10.2196/23252 (PMC7929744; doi:10.2196/23252)
Supplement: Multimedia Appendix 5 [file jmir_v23i2e23252_app5.pdf]

Impact on main clinical outcomes, significant and not significant effects.

Intervention	Design	Reference	HbA$_{1c}$	FBG	BP	Body weight	BMI	DR QoL	HR QoL	Cost-effective ness	Time saving	Other
**Not categorized**	**SR & MA**	(Mushcab et al. 2015)	+++									
		(Lee et al. 2018)	+++									
		(Greenwood et al. 2015)	+++									
		(Lee et al. 2017)	+++									
		(Zhai et al. 2014)	+++							1		
		(Jalil et al. 2015)										x
		(Kim et al. 2019)	+++				+++					
		(Kongstad et al. 2019)										x
		(Cassimatis/Kavanagh 2012)	+++									
		(Huang et al. 2015)	+++									
**"Real-time video"**	**RCT**	(Hansen et al. 2017)	++	0 (S N/R)	0^{sd} (S N/R)		0 (S N/R)		(0)			
		(Davis et al. 2010)	++		$(++)^{sd}$		(++)					
		(Rasmussen et al. 2016)	++	++	$(00)^{sd}$	++ (CG more weightloss)						
		(Toledo et al. 2014)	+, ++									
		(Egede et al. 2018)	0 (CG deterioration in values)									
		(Tavşanli et al. 2013)	++	(++)								
	**RCT, pilot**	(Dy et al. 2013)	++ (S N/R)									
		(Timmerberg et al. 2009)	(+) (greater, significant improve-ment n CG)									
	**Qualitative**	(Carlisle/Warren 2013)										x
		(Gordon et al.)									+ (S N/R)	
		(Robinson et al. 2016)										x

	**Non-randomized controlled trial**	(Ciemins et al. 2011)	(++)		(++)[sd]							
**“Real-time audio”**	**RCT**	(Benson et al. 2019)	(++)									
		(Vasconcelos et al. 2018)	+, (++)		(+)[sd], ++[s]		(+), (++)					
		(Barton et al. 2018)										x
		(Odnoletkova et al. 2016)	++			++	++					
		(Blackberry et al. 2013)	(++)									
		(Varney et al. 2016)								+ (S N/R)		
		(Varney et al. 2014)	+ (not sustained)	+ (not sustained)	+[d] (not sustained)							
		(Fernandes et al. 2016)										x
		(Trief et al. 2016)	+, (++)		++[d]		+, ++					
		(Goode et al. 2015)										x
		(Maslakpak et al. 2017)	(++)	(++)								
		(Sarayani et al. 2018)	+, (++)									
		(Schechter et al. 2016)								+ (S N/R)		
		(Swoboda et al. 2017)										x
		(Walker et al. 2011)	++									
	**Qualitative**	(Wu et al. 2010)										x
**“Real-time audio + asynchronous”**	**RCT**	(McMahon et al. 2012)	+, (++)		+[sd]	(-)	(-)					
**“Asynchronous”**	**RCT**	(Cho et al. 2017)	+, ++									
		(Ramadas et al. 2018)	+, (++)	+, (++)								
		(Egede et al. 2017)	++									
		(Tildesley et al. 2013)	+, (++)									
		(Cho et al. 2011)	+									
		(Avdal et al. 2011)	+									
		(Tildesley et al. 2010)	+, ++									
		(Lee et al. 2020)	(++)									

		(Wild et al. 2016)	++		++^{sd}							
		(Cho et al. 2011)	0 <6.5% (0) >6.5%								+	
		(Dario et al. 2017)	(++)						1			
		(Wakefield et al. 2011)	+, ++ (not sustained)		+^{s}, ++^{s}							
		(Wakefield et al. 2012)										x
		(Luley et al. 2011)	+, ++			+, ++	+, ++			+		
		(Kim/Kim 2008)	+									
		(Bentley et al. 2016)	++ (S N/R)			++ (S N/R)						
		(Goodarzi et al. 2012)	++									
		(Cho et al. 2009)	+, (++)									
		(Lim et al. 2016)	++				+					
		(Arora et al. 2014)	(++)									
		(Burner et al. 2018)	(+), (++)									
		(Fang/Deng 2018)	+, ++		(+)^{s} (significant improvement in CG)							
		(Fortmann et al. 2017)	++									
		(Del Prato et al. 2012)	(++)			0						
		(Kim et al. 2016)	+, ++	+								
	**Qualitative**	(Hanley et al. 2015)										x
		(Lee et al. 2019)										x
**"Combined" (real-time and asynchronous)**	**RCT**	(Parsons et al. 2019)	+, ++									
		(Carter et al. 2011)	+		(0)^{sd}		+					
		(Bujnowska-Fedak et al. 2011)	+, ++ (only among non-insulin-requiring patients)					(++)				
		(Jeong et al. 2018)	+, (++)	++								

		(Rodriguez-Idigoras et al. 2009)	+, (++)		+^{sd}		+					
		(Kempf et al. 2017)	++		++^{s}	++	++		++			
		(Wang et al. 2017)	+, ++									
		(Zhou et al. 2014)	+, ++	++	+^{sd}		(0)					
		(Chen et al. 2011)	+			(0)						
		(Nicolucci et al. 2015)	++		(++)^{sd}	(++)			++			
		(Pressman et al. 2014)	+, (++)		(++)^{s}	(++)	(++)					
		(Stone et al. 2010)	++		(++)^{sd}	(- -)						
		(Stone et al. 2012)	(++)									
		(Hsu et al. 2016)	+, ++			(++)					++<br>(S N/R)	
		(Wakefield et al. 2014)	(- -)		(++)^{s}							
		(Warren et al. 2018)	+, ++							++		
		(Castelnuovo et al. 2011)				(+),(++)						
		(von Storch et al. 2019)	+, ++				+, ++					
		(Liou et al. 2014)	++		(++)^{sd}		(++)					
		(Plotnikoff et al. 2013)	(- -)	(00)			(- -)					
	**RCT, pilot**	(Crowley et al. 2016)	++		++^{sd}							
	**Non-randomized controlled trial**	(McFarland et al. 2012)	++									
	**Cohort**	(Kesavadev et al. 2012)	+	+	+^{sd}		+			+<br>(S N/R)		
		(Jia et al. 2009)										x
	**Oberser-vational**	(Jha et al. 2016)	+	+, (++)				+				
		(Dienstl et al. 2011)	+	+	+^{sd}	+	+	+				
	**Non-controlled intervention study**	(Lewinski et al. 2019)			(0)^{s}							
	**Qualitative**	(Koopman et al. 2014)										x
		(Andrews et al. 2017)										x
		(Lee et al. 2018a)										x

**Subgroup “video clips”**	**RCT**	(Tang et al. 2013)	(++)		(++)^{sd}	(00)						
		(Greenwood et al. 2015)	++									
		(Steventon et al. 2014)	++									

x = study examined other than the selected outcomes
+++ = overall positive effects (SR/ MA)
++ = improvement in intervention group compared to control group (intergroup)
+ = improvement in intervention group compared to baseline (intragroup)
1 = inconclusive
000 = no overall effect (SR/MA)
00 = no changes in intervention group compared to control group (intergroup)
0 = no changes in intervention group compared to baseline (intragroup)
- - = deterioration in intervention group compared to control group (intergroup)
- = deterioration in intervention group compared to baseline (intragroup)
( ) = not statistically significant

Abbreviations:

BP = blood pressure; d = diastolic blood pressure; DL = diabetes-related; FBG = fasting blood glucose; HbA1c = hemoglobin A1c; HL = health-related; MA = meta-analysis; QoL = quality of life; RCT = randomized controlled trial; s = systolic blood pressure; S N/R = significance not reported; SR = systematic review; T2DM = type 2 diabetes mellitus
