# Supplementary material for: Effect of Telemetric Interventions on Glycated Hemoglobin A1c and Management of Type 2 Diabetes Mellitus: Systematic Meta-Review
Source: J Med Internet Res. 2021 Feb 17;23(2):e23252. doi: 10.2196/23252 (PMC7929744; doi:10.2196/23252)
Supplement: Multimedia Appendix 6 [file jmir_v23i2e23252_app6.pdf]

Significant effects on main clinical outcomes.

**Outcome/ Intervention**	**HbA1c**	**FBG**	**BP**	**Body weight**	**BMI**	**DRQoL**	**HRQoL**	**Cost**	**Time saving**
**SR & MA** (n=10)	(8/8) +				(1/1) +				
**"Real-time video"** (n=12)	(5/9)* +	(2/3) +		(1/1) +					
**"Real-time audio"** (n=17)	(6/9) +	(1/2) +	(3/3) +	(1/1) +	(1/3) +				
**"Asynchronous"** (n=28)	(17/24) +	(2/2) +	(2/3) +	(1/3) +	(2/2) +			(1/1) +	(1/1) +
**"Combined"** (n=30)	(21/24) +	(5/6) +	(6/13) +	(2/8) +	(6/10) +	(2/3) +	(2/2) +	(1/2) +	
**Sub group "Video clips"** (n=3)	(2/3) +								

* Number of studies with significant effects versus number of all studies (inclusive not sustained significant effects)
+ = improvement

Abbreviations:

N/S = not significant; SR = systematic review; MA = meta-analysis; HbA1c = hemoglobin A1c; FBG = fasting blood glucose; BP = blood pressure; DRQoL = diabetes-related quality of life; HRQoL = health-related quality of life
