# Supplementary material for: Effect of Telemetric Interventions on Glycated Hemoglobin A1c and Management of Type 2 Diabetes Mellitus: Systematic Meta-Review
Source: J Med Internet Res. 2021 Feb 17;23(2):e23252. doi: 10.2196/23252 (PMC7929744; doi:10.2196/23252)
Supplement: Multimedia Appendix 7 [file jmir_v23i2e23252_app7.pdf]

Changes in HbA_{1c} values (%) in intervention and control group from base line to end of the study (n=41 RCTs).

	Inter-vention duration	IG base line (MD)	IG base line (SD)	IG end (MD)	IG end (SD)	CG base line (MD)	CG base line (SD)	CG end (MD)	CG end (SD)	Mean difference (endpoints)
**"Real-time video interventions"**										
(Toledo et al. 2014)[21]	6	8.6	0.3	**6.6**	0.2	8.9	0.4	**8.1**	0.2	-1.5
(Tavşanli et al. 2013)[22]	6	7.79	0.78	**7.3**	0.79	7.92	2.22	**8.09**	1.28	-0.79
**Subtotal MD [95% CI]**										-1.15 [-1.84 to -0.45]
**Subtotal SD**										0.50
(Davis et al. 2010)[19]	12	9.4	0.3	**8.2**	0.4	8.8	0.3	**8.6**	0.3	-0.4
(Egede et al. 2018)[37]	12	6.9		**6.9**		7.3		**7.7**		-0.8
**Subtotal MD [95% CI]**										-0.6 [-0.99 to-0.31]
**Subtotal SD**										0.28
**"Real-time audio interventions"**										
(Vasconcelos et al. 2018)[32]	6	8	2.14	**7.21**	1.19	6.9	1.31	**7.33**	1.73	-0.12
(Odnoletkova et al. 2016)[25]	6	7	1.1	**6.8**	0.9	7	1	**7**	1.1	-0.2
(Varney et al. 2014)[33]	6	8.2		**7.7**		8.5		**8.5**		-0.8
**Subtotal MD [95% CI]**										-0.37 [-0.79 to 0.05]
**Subtotal SD**										0.38
**„Asynchronous interventions“**										
(Cho et al. 2011)[48]	3	8	0.8	**7.5**	0.9	8	1	**7.8**	1.1	-0.3
(Goodarzi et al. 2012)[42]	3	7.91	1.24	**7.02**	1.02	7.83	1.12	**7.48**	1.25	-0.46
**Subtotal MD [95% CI]**										-0.38 [-0.54 to -0.22]
**Subtotal SD**										0.11

	Inter-vention duration	IG base line (MD)	IG base line (SD)	IG end (MD)	IG end (SD)	CG base line (MD)	CG base line (SD)	CG end (MD)	CG end (SD)	Mean difference (endpoints)
(Cho et al. 2017)[36]	6	7.86	0.69	**7.55**	0.86	7.81	0.66	**7.7**	0.88	-0.15
(Ramadas et al. 2018)[55]	6	9.1	2	**8.7**	1.9	8.9	1.9	**8.3**	2.1	-0.4
(Lim et al. 2016)[43]	6	8	0.7	**7.3**	0.9	8.1	0.8	**7.9**	1.2	-0.6
(Fortmann et al. 2017)[45]	6	9.5	1.2	**8.5**	1.2	9.6	1.4	**9.4**	2	-0.9
(Kim et al. 2016)[46]	6	7.9	0.8	**6.7**	0.7	8	0.8	**7.4**	1.3	-0.7
(Avdal et al. 2011)[47]	6	8.008		**7.496**		8.141		**8.189**		-0.693
(Tildesley et al. 2010)[38]	6	8.8	1.3	**7.6**	0.74	8.5	1.2	**8.4**	1.1	-0.8
(Cho et al. 2011)[57]	6	8	0.8	**7.5**	0.9	8	1	**7.8**	1.1	-0.3
**Subtotal MD [95% CI]**										-0.57 [-0.75 to -0.39]
**Subtotal SD**										0.26
(Lee et al. 2020)[50]	12	9		**8.69**		9		**8.7**		-0.01
(Kim/Kim 2008)[49]	12	8.16	1.9	**6.67**	0.77	7.66	0.78	**8.19**	0.54	-1.52
**Subtotal MD [95% CI]**										-0.77 [-2.25 to 0.72]
**Subtotal SD**										1.07
**„Combined interventions“**										
(Kempf et al. 2017)[70]	3	8.4	1.3	**7.3**	1.1	8.2	1.2	**8**	1.3	-0.7
(Zhou et al. 2014)[64]	3	8.44	1.58	**6.84**	1.2	8.33	1.58	**7.6**	1.57	-0.76
(Hsu et al. 2016)[72]	3	10.8	1.2	**7.7**	1.6	10.9	1.2	**8.9**	2.2	-1.2
(Wakefield et al. 2014)[67]	3	7.2	0.21	**7.2**	0.2	7.4	0.18	**7.4**	0.18	-0.2
(von Storch et al. 2019)[73]	3	7.05	0.977	**6.58**	0.723	6.89	1.01	**6.95**	1.02	-0.37
**Subtotal MD [95% CI]**										-0.65 [-0.98 to -0.31]
**Subtotal SD**										0.39
(Crowley et al. 2016)[65]	6	10.5		**9.2**		10.5		**10.2**		-1
(Bujnowska-Fedak et al. 2011)[79]	6	6.95	0.82	**6.66**	0.86	7.21	2.02	**7.2**	1.86	-0.54
(Jeong et al. 2018)[58]	6	8.39	1.1	**7.44**	1.14	8.39	1.1	**7.7**	1.16	-0.26
(Wang et al. 2017)[80]	6	7.9		**6.8**	0.7	8		**7.3**	1.3	-0.5
(Stone et al. 2012)[71]	6	9.4		**8.71**	1.25	9.4		**8.84**	1.38	-0.13
(Warren et al. 2018)[75]	6	8.4		**7.5**		8.1		**8.1**		-0.6

	Inter-vention duration	IG base line (MD)	IG base line (SD)	IG end (MD)	IG end (SD)	CG base line (MD)	CG base line (SD)	CG end (MD)	CG end (SD)	Mean difference (endpoints)
(Liou et al. 2014)[66]	6	8.3	1.2	**7.6**	1.1	8.1	1.2	**8.1**	1.3	-0.5
**Subtotal MD [95% CI]**										-0.50 [-0.71 to -0.30]
**Subtotal SD**										0.28
(Parsons et al. 2019)[81]	12	8.6		**7.4**		8.7		**8.3**		-0.9
(Rodriguez-Idigoras et al. 2009)[59]	12	7.62	1.6	**7.4**	1.43	7.44	1.13	**7.35**	1.38	0.05
(Nicolucci et al. 2015)[69]	12	7.94	0.8	**7.44**	1	7.99	0.8	**7.78**	1.1	-0.34
(Plotnikoff et al. 2013)[82]	12	7.11		**7.28**		7.08		**7.07**		0.21
**Subtotal MD [95% CI]**										-0.25 [-0.73 to 0.24]
**Subtotal SD**										0.49
**Subgroup of „combined interventions“: „video clips“**										
(Tang et al. 2013)[76]	12	9.24	1.59	**8.1**	1.68	9.28	1.74	**8.33**	1.81	-0.23
(Steventon et al. 2014)[78]	12	8.38	1.68	**8.15**	1.49	8.41	1.64	**8.38**	1.6	-0.23
**Subtotal MD [95% CI]**										-0.23 [-0.23 to -0.23]
**Subtotal SD**										0

Abbreviations: CI = confidence interval, CG = control group, IG = intervention group, MD = mean deviation, RCT = randomized controlled trial, SD = standard deviation
