# Supplementary material for: Effect of Telemetric Interventions on Glycated Hemoglobin A1c and Management of Type 2 Diabetes Mellitus: Systematic Meta-Review
Source: J Med Internet Res. 2021 Feb 17;23(2):e23252. doi: 10.2196/23252 (PMC7929744; doi:10.2196/23252)
Supplement: Multimedia Appendix 8 [file jmir_v23i2e23252_app8.docx]

**Funnel plot using HbA_1c_ based on the RCTs from the subgroup meta-analysis.**
